# Not Too Young to Understand: Meaning Making From the Perspectives of School‐Aged Children With Cancer—A Scoping Review

**DOI:** 10.1002/pon.70592

**Published:** 2026-09-02

**Authors:** Yixuan Wang, Cheng Jin, Jennifer Currin‐McCulloch, David MacPhee, Abby R. Rosenberg, Yijia Yang, Zhenrong Su, Peichen Liu, Jiameng Wang, Sydney Pryor

**Affiliations:** ^1^ School of Social Work Colorado State University Fort Collins Colorado USA; ^2^ School of Social Work University of Minnesota Minneapolis Minnesota USA; ^3^ Department of Human Development and Family Studies Colorado State University Fort Collins Colorado USA; ^4^ Prevention Research Center Colorado State University Fort Collins Colorado USA; ^5^ Department of Supportive Oncology Dana‐Farber Cancer Institute Boston Massachusetts USA; ^6^ Department of Pediatrics Boston Children's Hospital Boston Massachusetts USA; ^7^ Department of Pediatrics Harvard Medical School Boston Massachusetts USA; ^8^ School of Social Work University of Connecticut Hartford Connecticut USA; ^9^ Suzanne Dworak‐Peck School of Social Work University of Southern California Los Angeles California USA; ^10^ School of Social Research Renmin University of China Beijing China; ^11^ Department of Psychology Colorado State University Fort Collins Colorado USA

**Keywords:** adaptation, cancer, child, meaning making, neoplasms, oncology, psychological

## Abstract

**Background:**

Meaning making is increasingly recognized as an important determinant of psychosocial adjustment in adult oncology. However, how school‐aged children make meaning of cancer and how developmental, relational, and sociocultural influences shape this process remain insufficiently characterized, particularly because frameworks are largely derived from adult research and centered on intrapersonal processes.

**Aims:**

To synthesize evidence on meaning making among school‐aged children with cancer and examine these findings through Park's meaning‐making model while identifying dimensions not fully captured by the model.

**Methods:**

A scoping review was conducted according to PRISMA‐ScR guidelines. Four databases and reference lists were searched for peer‐reviewed studies examining meaning making through direct reports from children aged 6–12 years with cancer. Park's meaning‐making model informed study selection, data extraction, and deductive coding, while inductive coding identified dimensions not represented in the model.

**Results:**

Thirty‐nine studies were included. Children's meaning making encompassed global beliefs and developmental and healthcare‐related goals; appraisals of threats to the self, normalcy, relationships, and future; and cognitive, emotional, socially informed, spiritual, experiential, and avoidance‐related processes. Meaning making emerged as a dynamic, developmentally grounded, and relational process shaped by caregivers, healthcare professionals, and sociocultural contexts across the cancer trajectory. Evidence regarding children's sense of meaning or purpose and meanings made was limited.

**Conclusions:**

School‐aged children actively construct meaning throughout the cancer trajectory. Integrating interdisciplinary support for meaning making that is child‐centered and developmentally and culturally responsive into psychosocial care, serious illness communication, and pediatric palliative care may strengthen children's participation, coping, and psychosocial–spiritual well‐being.

## 1 Background

In the United States, pediatric cancer incidence has risen over recent decades, with nearly 15,000 children and adolescents diagnosed annually [1]. Advances in treatment and supportive care have substantially improved survival, contributing to a rapidly growing population of childhood cancer survivors that is projected to exceed 580,000 by 2040 [1]. However, cancer and its treatment can disrupt children's physical, neurocognitive, and socioemotional functioning, existential well‐being, and developmental trajectories, placing them at heightened risk for adverse psychosocial–spiritual outcomes [2, 3, 4, 5, 6]. These potential consequences underscore the importance of identifying processes that shape children's adjustment and may offer opportunities for support during critical periods of development.

Although the objective circumstances of cancer and treatment influence children's adjustment, their effects also depend on how children interpret and make meaning of these experiences. Children with cancer face numerous stressors that cannot be readily controlled, including an unpredictable disease course, intensive treatment demands, persistent symptoms and long‐term side effects, disruptions to daily routines and social relationships, and concerns about mortality [7, 8, 9, 10]. Because these stressors often cannot be changed, adjustment may hinge not only on managing their effects but also on reconciling cancer with children's evolving understandings of themselves, their lives, and their futures. Meaning making may therefore represent an important process through which children adapt to cancer, with implications for their psychosocial–spiritual adjustment and broader quality of life. Yet meaning making remains underexamined in children, particularly school‐aged children, who are often considered “too young to understand.”

This assumption may overlook school‐aged children's existing and developing capacities to understand serious illness and derive meaning from it. School‐aged children ages 6 to 12 undergo advances in reasoning, perspective taking, and self‐regulation [11, 12] that support increasingly sophisticated understandings of illness and the construction of meaning from adverse experiences. Importantly, the ways children interpret illness vary within this group and cannot be inferred from chronological age alone. Rather, these interpretations are shaped by children's cognitive and socioemotional capacities, as well as lived experiences such as prior illness experience and communication with adults [13, 14]. Beyond these individual differences, children's meaning making is inherently relational and embedded within family, peer, school, digital, and broader sociocultural contexts as children engage in social comparison, internalize cultural norms, and develop their sense of self [15, 16].

Within pediatric oncology and palliative care, children's meaning making may be co‐constructed within families and further shaped through communication with healthcare providers as children and families navigate shared, uncontrollable illness‐related stressors [17]. Parents may help children interpret illness through shared family narratives, while providers can support understanding by offering developmentally appropriate information [18]. Embedded within healthcare and broader sociocultural contexts [8, 15], these relational processes are central to pediatric palliative care, which supports communication among children, families, and providers about illness, uncertainty, goals of care, and quality of life across the cancer trajectory [19, 20]. Yet, children often have limited opportunities to participate in health‐related conversations, potentially marginalizing their voices within care partnerships [21]. Consequently, children's voices remain underrepresented in patient‐reported outcome measures, particularly in meaning‐related domains, reflecting continued reliance on adult proxies rather than direct child engagement [22]. These gaps underscore the need for child‐centered inquiry that situates meaning making within the developmental, relational, and contextual conditions under which it unfolds.

### 1.1 Park's Meaning‐Making Model

According to Park [23], meaning making occurs through the dynamic interplay between global meaning, which comprises an individual's beliefs, goals, and subjective sense of meaning or purpose, and situational meaning. Situational meaning encompasses the appraised meaning of a potentially stressful event, discrepancies between appraised and global meaning, subsequent meaning‐making processes, and meanings made. Within this framework, meaning making is typically distress driven, as discrepancies between appraised and global meaning may generate distress and prompt coping efforts to manage the demands of the situation. Coping may include problem‐focused strategies directed toward changing the stressor and emotion‐focused strategies directed toward managing emotional responses to it [24]. When a stressor is chronic or largely uncontrollable, meaning‐making coping may address discrepancies by revising appraised meaning, global meaning, or both [25, 26]. These coping responses may overlap rather than operate independently [25].

**FIGURE 1:**
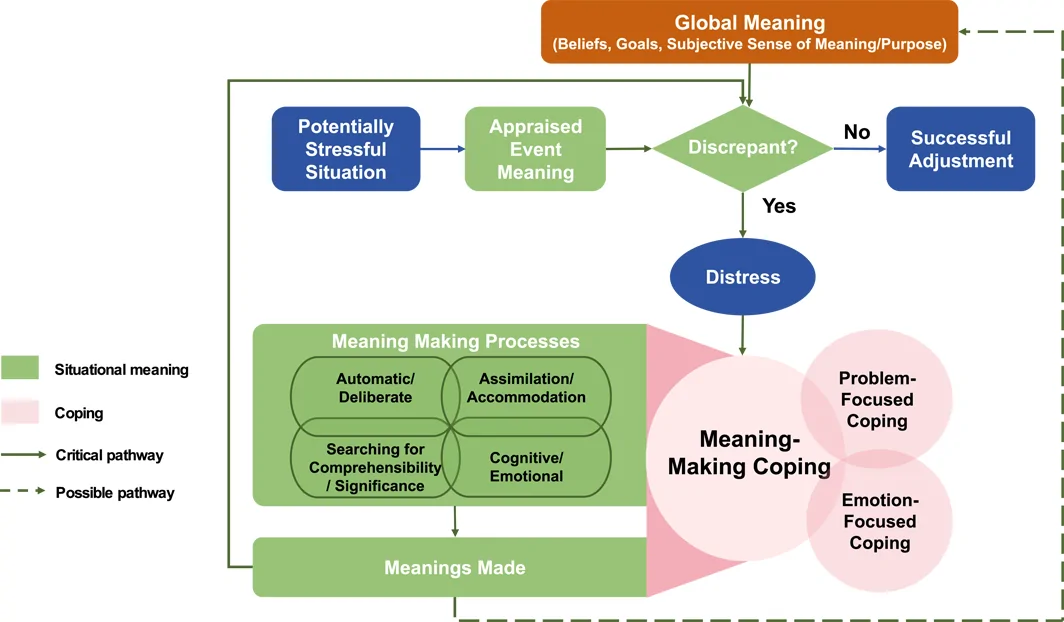
Conceptual pathways linking meaning making, coping, and adjustment. Developed by the authors based on Park's meaning‐making model [23], which builds on and further develops the earlier framework proposed by Park and Folkman [26]. The representation of related coping responses was informed by Lazarus and Folkman [24] and Folkman and Moskowitz [25]. Overlapping areas indicate that coping responses and meaning‐making dimensions are not mutually exclusive.

Meaning‐making coping comprises *meaning‐making processes*, which involve efforts to reduce meaning discrepancies, and *meanings made*, which represent the outcomes of those efforts [23, 26]. Meaning‐making processes may operate automatically or deliberately and may be assimilative or accommodative, oriented toward comprehensibility or significance, and cognitive or emotional; these dimensions may also overlap [23]. Meaning‐focused coping, for example, is a deliberate process that may involve both cognitive and emotional efforts [25]. Meanings made may support adjustment when they reduce discrepancies between appraised and global meaning [23]. If discrepancies and distress persist, meaning‐making processes may continue or recur, making the pathway iterative rather than linear [23]. Although conceptually distinguishable, global meaning, appraised meaning, meaning‐making processes, and meanings made are dynamically interrelated, and a single account or interpretation of an experience may reflect multiple components. Table 1 summarizes key concepts and relationships across meaning making, coping, and adjustment, while Figure 1 depicts their overlapping relationships and iterative pathways.

**TABLE 1:** Key concepts and relationships across meaning making, coping, and adjustment.

Conceptual grouping	Core concept	Definition and examples
Global meaning	Beliefs	Broad beliefs about the world and oneself, including justice, control, predictability, and coherence, that guide the interpretation of experience.
	Goals	Internal representations of desired processes, events, or outcomes that provide direction and motivation.
	Subjective sense of meaning or purpose	A feeling of meaningfulness and a sense of purpose or direction derived from viewing one's actions as oriented toward a desired future state or goal.
Situational meaning	Appraised meaning	The meaning assigned to a particular person–environment encounter, including its personal significance, threat, controllability, perceived causes, and implications for the future.
	Meaning discrepancy and distress	A perceived mismatch between an event's appraised meaning and one's global beliefs, goals, or sense of purpose. Such discrepancies may generate distress and prompt coping or meaning‐making efforts.
Related coping responses	Meaning‐making coping	Coping directed toward reducing discrepancies and restoring coherence by changing appraised meaning, global meaning, or both. It encompasses meaning‐making processes and the meanings made as outcomes of those efforts.
	Problem‐focused coping	Efforts directed toward addressing or modifying the problem causing distress.
	Emotion‐focused coping	Efforts directed toward managing or alleviating the emotional distress associated with the problem.
Components of meaning‐making coping	Meaning‐making processes	Processes through which individuals attempt to reduce discrepancies between appraised and global meaning. These processes may vary across four overlapping dimensions.
	Automatic or deliberate	Automatic processes occur without conscious intention and may include intrusive thoughts or avoidance of reminders. Deliberate processes involve intentional meaning‐related efforts. Meaning‐focused coping is one deliberate form and may involve both cognitive and emotional efforts.
	Assimilative or accommodative	Assimilation involves revising the event's appraised meaning to align with existing global meaning, whereas accommodation involves modifying global beliefs or goals to incorporate the experience.
	Comprehensibility or significance	Searching for comprehensibility involves seeking to understand how or why an event occurred, whereas searching for significance concerns what the event means for one's identity, goals, and life.
	Cognitive or emotional	Cognitive processing involves reflecting on and reappraising an experience in relation to existing beliefs and goals, whereas emotional processing involves experiencing, identifying, expressing, and reflecting on the emotions evoked by the experience.
	Meanings made	Products or changes resulting from attempts to reduce discrepancies between appraised and global meaning, including having made sense of the event, acceptance, revised causal understanding (reattribution), perceived growth or positive life changes (benefit finding), identity change, reappraisal of the stressor, changes in global beliefs or goals, and a restored or changed sense of meaning in life.
Downstream outcome	Adjustment	Adaptation following a stressful event, which may improve as discrepancies between appraised and global meaning are reduced.

*Note:* Meaning‐making concepts and definitions were informed by Park's model [23], which builds on and further develops the earlier framework proposed by Park and Folkman [26]. Definitions of related coping responses were informed by Lazarus and Folkman [24] and Folkman and Moskowitz [25]. Meaning‐making dimensions and coping responses may overlap rather than operate independently.

Park's meaning‐making model is based largely on adult research and may not fully capture developmental processes and capacities in childhood. Although Park [23] acknowledged that relational and sociocultural contexts may shape global and situational meaning, their influence is not explicitly integrated into the model's primarily intrapersonal structure. In the absence of a theory that specifically explains meaning making among children with cancer, this review uses Park's model as a conceptual guide while remaining attentive to developmental differences, relational and sociocultural influences, and children's lived experiences.

## 2 Method

### 2.1 Search Strategy

This scoping review followed the Preferred Reporting Items for Systematic Reviews and Meta‐Analyses Extension for Scoping Reviews (PRISMA‐ScR) and examined empirical evidence on meaning making from the perspectives of school‐aged children with cancer. In March 2025, the lead author systematically searched four databases: APA PsycInfo, CINAHL, MEDLINE, and Web of Science. To identify child‐focused literature while minimizing retrieval of adult‐focused studies, a proximity‐based search strategy was used (Supporting Information S1). Specifically, child‐related search terms and subject headings were linked within three words to both meaning‐making terms and child‐perspective terms (e.g., “child* N3 meaning*”). This strategy was designed to balance sensitivity and specificity by linking age‐based terms to meaning‐making constructs and child‐reported data, consistent with evidence synthesis guidance [27, 28]. To identify studies potentially missed by the proximity‐based search, primary studies cited in review articles identified during the initial screening process were also screened.

### 2.2 Eligibility Criteria

Eligibility criteria were defined a priori to identify studies examining meaning making from children's own perspectives in pediatric oncology. Eligible studies were peer‐reviewed empirical articles published in English that (1) included children aged 6–12 years at participation; (2) examined meaning making related to cancer; and (3) used children's direct reports or creative outputs, such as interviews, drawings, or photographs, as a primary data source. Studies were eligible when their findings could be conceptually mapped onto one or more of four model dimensions: global meaning, appraised meaning and associated distress, meaning‐making processes, and meanings made, even if the authors did not explicitly use meaning‐making terminology. Table 2 presents the conceptual prompts used during screening to operationalize this criterion. Reviews, conference abstracts, book chapters, editorials, commentaries, dissertations, theses, and protocols were excluded. Studies were also excluded if they did not report separate findings for children aged 6–12 years with cancer, focused solely on psychological assessments or symptom reports without addressing meaning making, or relied exclusively on proxy reports.

**TABLE 2:** Conceptual screening prompts guided by the meaning‐making model.

Meaning‐making dimension	Screening prompts
Global meaning	How do children with cancer describe their beliefs about health, illness, death and dying, themselves, and others; their developmental and health‐related goals (i.e., desired processes, events, or outcomes); and their broader sense of meaning, purpose, or direction in life?
Appraised meaning and associated distress	What meanings do children assign to cancer‐ and treatment‐related experiences? How do these appraisals shape whether and why the experiences are perceived as distressing?
Meaning‐making processes	How do children process or cope with cancer‐ and treatment‐related stress through meaning making? Do these processes occur automatically or deliberately, involve assimilation or accommodation, seek comprehensibility or significance, or involve cognitive or emotional processing?
Meanings made	Where longitudinal data are available: Do children demonstrate changes over time consistent with meanings made, such as acceptance, reattribution, perceived growth or positive life changes, changes in identity, revised beliefs or goals, or a restored sense of meaning or purpose?

### 2.3 Study Selection

Seven reviewers with backgrounds in social work, developmental science, and psychology participated in study selection. Before screening, the lead author provided standardized training on the meaning‐making model and predefined eligibility criteria. Reviewers then completed a pilot calibration exercise using a subset of records, compared their decisions, and discussed differences to clarify the criteria. Following deduplication, selection proceeded in two stages. First, titles and abstracts were independently screened by at least two reviewers. Second, each potentially eligible full‐text article was independently assessed by at least two reviewers using the predefined criteria and conceptual prompts in Table 2. At both stages, discrepancies were resolved through group discussion during reviewer meetings until consensus was reached. Rayyan [29] and Microsoft Excel were used to manage records and document screening decisions and reasons for exclusion.

### 2.4 Data Extraction and Analysis

Data extraction and analysis were guided by Park's [23] meaning‐making model and informed by methodological recommendations for scoping reviews [30]. A combined deductive–inductive approach was used, with themes iteratively identified, interpreted, and refined. Deductive coding drew on the model's concepts, whereas inductive coding was used to interpret developmental, relational, sociocultural, and illness‐specific features of pediatric cancer experiences within the included studies that were not fully represented in the model. Because the model conceptualizes meaning making as reducing discrepancies between appraised and global meaning, the same cancer‐related experience could be coded in different domains depending on its analytic function. For example, information could represent a healthcare‐related goal when desired, a threat to the self when withheld, or a meaning‐making process when actively sought.

Descriptive study characteristics were independently extracted by seven reviewers, with each study extracted by two reviewers to support accuracy. The lead author then extracted findings relevant to meaning making and conducted initial conceptual coding across four model‐derived domains: global meaning, appraised meaning and associated distress, meaning‐making processes, and meanings made. The second author independently applied the resulting analytical framework and checked the coding against the source articles to assess completeness and identify differences in code assignment or interpretation. Because coding was iterative and overlapping codes were permitted, neither interrater reliability nor percentage agreement was calculated. Instead, the first and second authors compared their coding, resolved differences through discussion, and documented final decisions and framework refinements in a decision log. Two senior researchers (the third and fourth authors), with expertise in oncology social work and child development, independently reviewed the synthesized findings for conceptual and analytical rigor and developmental alignment. Any additional interpretive differences were discussed and resolved.

## 3 Results

### 3.1 Screening Outcomes

**FIGURE 2:**
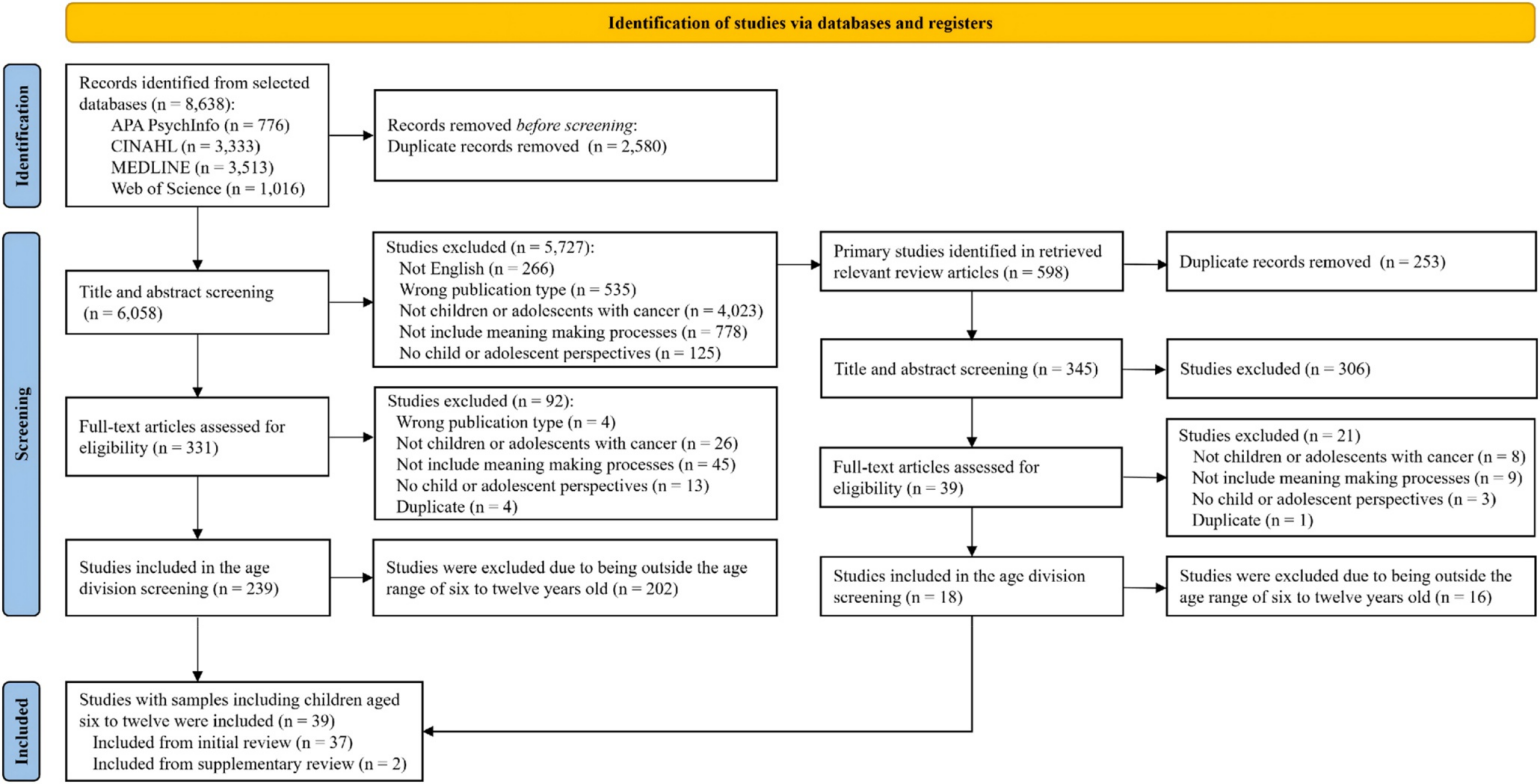
Preferred reporting of items for systematic reviews and meta‐analyses (PRISMA) flow diagram.

The initial search identified 8368 records. After 2580 duplicates were removed, 6058 titles and abstracts were screened, and 331 full‐text articles were assessed. Of these, 239 met the preliminary criteria and underwent further age‐specific assessment; 37 met all eligibility criteria. Supplementary reference screening identified 598 additional records and yielded two eligible studies. Overall, 39 studies were included in the review (Figure 2).

### 3.2 Characteristics of Included Studies

Study designs and sample characteristics are summarized in Table 3. Studies were published between 1990 and 2024, with over half published since 2015 (*n* = 20, 51.3%). Studies spanned diverse geographic and sociocultural contexts, with most conducted in North America (*n* = 13, 33.3%) and Europe (*n* = 12, 30.8%), followed by South America (*n* = 6, 15.4%), the Middle East (*n* = 4, 10.3%), and East, South, and Southeast Asia (*n* = 4, 10.3%). Research most frequently examined children's understanding of illness and treatment (*n* = 11, 28.2%) and their physical or psychosocial functioning and needs (*n* = 8, 20.5%). Fewer studies addressed coping (*n* = 4, 10.3%), schooling (*n* = 3, 7.7%), interactions with healthcare professionals (*n* = 3, 7.7%), sense of self (*n* = 2, 5.1%), and perceptions of death (*n* = 1, 2.6%). Four studies (10.3%) evaluated interventions, including arts‐based and technology‐assisted approaches, and one (2.6%) examined ethical dilemmas. Only three studies (7.7%) explicitly examined the meanings children assigned to cancer‐related experiences [34, 41, 53], and none was grounded in a defined meaning‐making framework.

**TABLE 3:** Study designs and sample characteristics of included studies (*N* = 39).

Author(s), year	Country	Aim	Study design	Sample type and size	Age Range (Years)	Sex or gender	Time since diagnosis	Cancer type	Treatment type	Disease course	Child meaning‐making data source	Child participation role
Ebrahimpour & Mirlashari, 2024 [31]	Iran	To examine how children living with leukemia perceive hope.	Cross‐sectional qualitative study (descriptive)	Children only: *N* = 20	6–12 (Mean = 8.9, SD = 2.2)	Female: *N* = 12 Male: *N* = 8	1 month to 6 years	Leukemia	Not specified	Newly diagnosed, active treatment, off treatment, relapse, and recovery	Drawing and semi‐structured interview	Data collection, member checking
Petronio‐DeFanti & Schwartz‐Barcott, 2024 [32]	United States	To explore how hospitalized children with cancer perceive approachable nurses and the impact of such nurses.	Cross‐sectional qualitative study (descriptive)	Children only: *N* = 7	8–12 (Mean = 10.6, SD = 1.6)	Female: *N* = 4 Male: *N* = 3	Not specified	Osteogenic sarcoma, acute lymphocytic leukemia, non‐Hodgkin's lymphoma, t‐cell lymphoma, and alveolar rhabdomyosarcoma	Not specified	Not specified	Semi‐structured interview with drawing aid	Data collection
Dinç et al., 2023 [33]	Turkey	To examine the hospital classroom experience of school‐aged children with cancer.	Cross‐sectional qualitative study (descriptive)	Children only: *N* = 17	7–12 (Mean = 8.9, SD = 1.5)	Female: *N* = 8 Male: *N* = 9	Not specified	Acute myeloid leukemia, acute lymphoblastic leukemia	Not specified	Not specified	Semi‐structured interview	Data collection
von Werthern et al., 2023 [34]	United Kingdom	To explore children's experiences with completing treatment for acute lymphoblastic leukemia, how endings were signified and marked, as well as the meaning that is given to it.	Cross‐sectional qualitative study (phenomenology)	Children only: *N* = 7	7–12	Female: *N* = 5 Male: *N* = 2	Not specified	Acute lymphoblastic leukemia, pre‐b acute lymphoblastic leukemia	Chemotherapy	Off treatment	Semi‐structured interview with drawing aid	Study protocol design, data collection, member checking
Lee Wong et al., 2022 [35]	China	To assess the feasibility and acceptability of using immersive virtual reality to manage anxiety, nausea, and vomiting in pediatric cancer patients receiving their first chemotherapy.	Mixed‐methods randomized control trial (exploratory design)	*N* = 47: Pediatric patients (*n* = 19), accompanying parents (*n* = 19), nurses (*n* = 9)	6–12 intervention group (Mean = 10.33, SD = 1.50); control group (Mean = 9.11, SD = 1.60)	Female: *N* = 7 Male: *N* = 12	Not specified	Leukemia, lymphoma, bone tumor	Chemotherapy	Active treatment	Semi‐structured interview	Intervention, data collection
Linder et al., 2022 [36]	United States	To summarize children's self‐reported symptoms and daily experiences during the feasibility and acceptability trial of a game‐based app.	Mixed‐methods descriptive study (convergent design)	Children only: *N* = 20	6–12 (Mean = 8.7, SD = 1.9)	Female: *N* = 7 Male: *N* = 13	1–74 months (Mean = 12.1, SD = 20.1)	Acute lymphoblastic leukemia, brain tumor, Hodgkin's lymphoma, non‐Hodgkin's lymphoma, osteosarcoma	Chemotherapy	Active treatment, relapse	App‐entered data through the symptom tracking feature, daily goal page, diary, and sketch pad	Co‐designed intervention, data collection
Aşikli & Aydin Er, 2021 [37]	Turkey	To determine the definitions of a good physician and a good nurse provided by elementary school‐age oncology patients.	Cross‐sectional qualitative study (grounded theory)	Children only: *N* = 18	7–12 (Mean = 9.6, SD = 1.8)	Female: *N* = 6 Male: *N* = 12	0.5–42 months (Mean = 6, SD = 9.3)	Not specified	Not specified	Active treatment	Semi‐structured interview with drawing aid	Data collection
Ebrahimpour et al., 2021 [38]	Iran	To identify the views of hospitalized children with cancer regarding the circumstances and factors that foster hope in the ward.	Cross‐sectional qualitative study (descriptive)	Children only: *N* = 20	6–12 (Mean = 9.1, SD = 2.1)	Female: *N* = 10 Male: *N* = 10	1–72 months (Mean = 13.5, SD = 15.8)	Acute lymphoblastic leukemia, Ewing's sarcoma, abdominal malignancy, lymphoma	Chemotherapy	Active treatment, relapse	Semi‐structured interview using photovoice	Data collection, member checking
Petronio‐Coia & Schwartz‐Barcott, 2020 [39]	United States	To examine how hospitalized children perceive approachability in interactions with nurses on oncology units.	Cross‐sectional qualitative study (descriptive)	Children only: *N* = 7	8–12 (Mean = 10.6, SD = 1.6)	Female: *N* = 4 Male: *N* = 3	Not specified	Osteogenic sarcoma, acute lymphocytic leukemia, non‐Hodgkin's lymphoma, t‐cell lymphoma and alveolar rhabdomyosarcoma	Not specified	Not specified	Semi‐structured interview with drawing aid	Data collection
Boles & Winsor, 2019 [40]	United States	To examine how children with cancer represent their school and illness experiences across developmental domains using drawings.	Cross‐sectional qualitative study (phenomenology)	Children only: *N* = 10	6–11 (Mean = 8.63)	Female: *N* = 4 Male: *N* = 6	Not specified	Solid tumor, leukemia	Not specified	Active treatment	Interview‐elicited drawings	Data collection
Fernandes & Souza, 2019 [41]	Brazil	To reveal if the theme of death itself or of other children is addressed in the drawing stories created by children to understand the meaning of childhood cancer.	Cross‐sectional qualitative study (descriptive)	Children only: *N* = 5	6–11 (Mean = 8, SD = 2.1)	Female: *N* = 4 Male: *N* = 1	Not specified	Ovarian cancer, sarcoma, osteosarcoma, acute lymphoid leukemia	Not specified	Active treatment	Drawing‐and‐story procedure	Data collection
Mant et al., 2019 [42]	United Kingdom	To understand what it feels like for a child to be told, “you have cancer.”	Longitudinal qualitative study (phenomenology)	Children only: *N* = 6	8–12 (Mean = 9.7, SD = 1.9)	Female: *N* = 4 Male: *N* = 2	8–70 days in the first interview (Mean = 33.5, SD = 24.0) 19–86 days in the second interview (Mean = 50.8, SD = 24.1)	Hodgkin's lymphoma, pilocytic astrocytoma, t‐cell non‐Hodgkin's lymphoma, acute lymphoblastic leukemia, acute myeloid leukemia	Not specified	Newly diagnosed, active treatment	Semi‐structured interview using draw and write techniques	Data collection
Emidio, 2018 [43]	Brazil	To understand how children perceive the treatment they receive in a pediatric oncology unit.	Cross‐sectional qualitative study (descriptive)	Children only: *N* = 5	6–12	Female: *N* = 3 Male: *N* = 2	Not specified	Acute lymphoid leukemia, acute myeloid leukemia, rhabdomyosarcoma	Not specified	Not specified	Semi‐structured interview	Data collection
França et al., 2018 [44]	Brazil	To understand the existential experience of children with cancer under palliative care based on humanistic nursing theory.	Cross‐sectional qualitative study (descriptive)	Children only: *N* = 11	7–11 (Mean = 9.5, SD = 1.8)	Female: *N* = 5 Male: *N* = 6	7 months to 2 years	Leukemia, lymphoma, osteosarcoma, nephroblastoma or Wilms' tumor and ovarian cancer	Chemotherapy, radiation therapy, surgery	Palliative phase	Story drawing procedure	Data collection
Linder et al., 2018 [45]	United States	To describe how school‐aged children with cancer represent their symptoms and associated characteristics using draw‐and‐tell interviews.	Cross‐sectional qualitative study (descriptive)	Children only: *N* = 27	6.33–12.83 (Mean = 9.16, SD = 1.9)	Female: *N* = 13 Male: *N* = 14	1–93 months (median = 9)	Acute lymphoblastic leukemia, sarcoma, brain tumor, Hodgkin lymphoma, non‐Hodgkin lymphoma, other acute leukemia, other solid tumor	Not specified	Active treatment, relapse	Draw‐and‐tell interview	Data collection
Linder et al., 2017 [46]	United States	To characterize the “good days” and “sick days” of elementary school‐age children through their drawings.	Cross‐sectional qualitative study (descriptive)	Children only: *N* = 27	6.33–12.83 (Mean = 9.16, SD = 1.9)	Female: *N* = 13 Male: *N* = 14	1–93 months (Mean = 15.5, SD = 17.7 median = 9)	Acute lymphoblastic leukemia, sarcoma, brain tumor, Hodgkin lymphoma, non‐Hodgkin lymphoma, other acute leukemia, other solid tumor	Chemotherapy	Active treatment, relapse	Draw‐and‐tell interview	Data collection
Xie et al., 2016 [47]	China	To describe the experiences and nursing needs of school‐age Chinese children undergoing lumbar punctures for acute lymphoblastic leukemia.	Cross‐sectional qualitative study (descriptive)	Children only: *N* = 21	7–12	Female: *N* = 9 Male: *N* = 12	0–3 months	Acute lymphoblastic leukemia	Not specified	Active treatment	Semi‐structured interview	Data collection, member checking
Einberg et al., 2015 [48]	Sweden	To explore what promotes health from the perspective of children with experience undergoing cancer treatment.	Cross‐sectional qualitative study (descriptive)	Children only: *N* = 15	8–12 (Mean = 10.4, SD = 1.3)	Female: *N* = 5 Male: *N* = 10	Over 4 years	Acute lymphoblastic leukemia, acute myeloid leukemia, non‐Hodgkin's lymphoma	Chemotherapy	Off treatment (majority)	Photography and semi‐structured focus group interview	Data collection
Lima & Santos, 2015 [49]	Brazil	To understand the influence of play on the care process as perceived by children with cancer.	Cross‐sectional qualitative study (descriptive)	Children only: *N* = 8	6–12 (Mean = 8.5, SD = 2.4)	Female: *N* = 2 Male: *N* = 6	22–84 months (Mean = 49.6, SD = 22.5)	Leukemia, medulloblastoma	Chemotherapy, consolidation therapy	Active treatment (maintenance), relapse	Photography and semi‐structured interview	Data collection
Sposito et al., 2015 [50]	Brazil	To analyze the coping strategies used by children undergoing chemotherapy for cancer during hospitalization.	Cross‐sectional qualitative study (descriptive)	Children only: *N* = 10	7–12 (Mean = 9.8, SD = 1.93)	Female: *N* = 5 Male: *N* = 5	4–33 months (Mean = 11.4, SD = 8.51)	Osteosarcoma, acute lymphoid leukemia, non‐Hodgkin's lymphoma, Ewing's sarcoma, rhabdomyosarcoma, medulloblastoma	Chemotherapy	Active treatment	Semi‐structured interview using puppet aid	Data collection
Einberg et al., 2014 [51]	Sweden	To describe how children undergoing cancer treatment perceive friendship.	Cross‐sectional qualitative study (descriptive)	Children only: *N* = 15	8–12 (Mean = 10.4, SD = 1.3)	Female: *N* = 5 Male: *N* = 10	Not specified	Acute lymphoblastic leukemia, acute myeloid leukemia, non‐Hodgkin's lymphoma	Not specified	Not specified	Semi‐structured focus group interview	Data collection
Hildenbrand et al., 2014 [52]	United States	To describe how children cope with cancer‐related stressors during treatment and how parents assist this coping.	Mixed‐methods descriptive study (convergent design)	*N* = 32: Children (*n* = 15) and their parents (*n* = 17)	6–12 (Mean = 9.47, SD = 1.99)	Not specified	1 day to 27 weeks (Mean = 6.8 weeks, SD = 8.63 weeks)	Acute lymphoblastic leukemia, brain tumor, lymphoma, osteosarcoma, other cancers	Not specified	Active treatment	Semi‐structured interview, how I coped under Pressure Scale	Data collection
Sousa et al., 2014 [53]	Brazil	To understand the meaning of experiencing illness for children with cancer.	Cross‐sectional qualitative study (descriptive)	Children only: *N* = 8	6–12	Female: *N* = 3 Male: *N* = 5	8 months to 6 years	Leukemia, lymphoma, kidney cancer, ovarian cancer	Chemotherapy	Active treatment, off treatment	Thematic drawing‐story technique	Data collection
Ångström‐Brännström et al., 2013 [54]	Sweden	To describe a child's experience of receiving care until death, focusing on both discomfort and comfort.	Longitudinal qualitative study (case study)	*N* = 3: Child (*n* = 1), mother (*n* = 1), and nurse (*n* = 1)	9	Male: *N* = 1	Not specified	Not specified	Chemotherapy	Active treatment, relapse, palliative phase, end‐of‐life stage	Drawing, interview	Data collection
Sadruddin & Hameed‐ur‐Rehman, 2013 [55]	Pakistan	To explore the self‐perception of children with cancer and examine the relationship between children and others.	Cross‐sectional qualitative study (phenomenology)	Children only: *N* = 78	7–12	Female: *N* = 43 Male: *N* = 35	Not specified	Not specified	Not specified	Active treatment (first stage)	Drawing	Data collection
Rodgers et al., 2012 [56]	United States	To identify anticipatory, acute, and delayed chemotherapy‐induced nausea and vomiting (CINV) frequencies, as well as the coping strategies used, among pediatric patients with cancer.	Prospective quantitative study (descriptive, cohort design)	Children only: *N* = 40	7–12 (Mean = 9.6, SD = 1.7)	Female: *N* = 8 Male: *N* = 32	Not specified	Leukemia, solid tumor, lymphoma, medulloblastoma	Chemotherapy, radiation therapy, bone marrow transplantation	Active treatment	Kidcope–Younger Version	Data collection
Hildenbrand et al., 2011 [57]	United States	To explore common cancer‐related stressors experienced by children, as well as how children cope with these stressors and how parents assist child coping during treatment.	Cross‐sectional qualitative study (descriptive)	*N* = 30: Children (*n* = 15), caregiver (*n* = 15)	6–12 (Mean = 8.8, SD = 1.7)	Female: *N* = 7 Male: *N* = 8	1 week to 3.5 years (Mean = 49 weeks, SD = 53.8 weeks)	Leukemia, brain tumors, lymphoma, neuroblastoma, sarcomas	Not specified	Active treatment	Semi‐structured interview	Data collection
Nguyen et al., 2010 [58]	Vietnam	To evaluate whether music therapy affects pain and anxiety in children undergoing lumbar punctures.	Mixed‐methods randomized control trial (exploratory design)	Children only: *N* = 40	7–12 intervention group (Mean = 8.8, SD = 1.59); control group (Mean = 9.4, SD = 1.93)	Female: *N* = 15 Male: *N* = 25	Not specified	Leukemia	Not specified	Not specified	Interview with open‐ended questions	Intervention, data collection
Vatne et al., 2010 [59]	Norway	To explore how children with and without cancer understood symptom terms, including perceived causes, consequences, cures, and the language they used to describe symptoms.	Cross‐sectional qualitative study (descriptive)	*N* = 14: Children with cancer (*n* = 6), healthy children (*n* = 8)	8–12	Female: *N* = 4 Male: *N* = 2	Not specified	Leukemia, soft tissue tumors	Not specified	Active treatment, end of treatment	Semi‐structured interview involving the creation of a fictive person with a name and the same age, sex, and situation in life as the interviewee	Study protocol, data collection
Enskär & von Essen, 2008 [60]	Sweden	To describe physical diseases, treatment‐related problems, and psychosocial functioning among children with cancer.	Cross‐sectional quantitative study (descriptive)	Children only: *N* = 39	7–12 (Mean = 9.6, SD = 1.55)	Female: *N* = 16 Male: *N* = 23	Less than a year to over 3 years	Leukemia, lymphoma, brain tumors, and other tumors	Not specified	Not specified	The life situation Scale for children and an additional open‐ended question	Data collection
Horstman et al., 2008 [61]	United Kingdom	To explore children with cancer's perceptions of their care and support needs, including their positive and negative experiences with cancer care services.	Cross‐sectional qualitative study (descriptive)	Children only: *N* = 17	6–12 (Mean = 9.6, SD = 1.55)	Female: *N* = 11 Male: *N* = 6	Not specified	Not specified	Not specified	Active treatment, end of treatment, off treatment	Semi‐structured interview using draw‐and‐write technique	Data collection
Fredman et al., 2007 [62]	United Kingdom	To address ethical dilemmas in clinical pediatric care.	Cross‐sectional qualitative study (case report)	Children only: *N* = 1	10	Female: *N* = 1	Not specified	Bone cancer	Surgery, radio therapy and chemotherapy	Not specified	N/A	N/A
Björk et al., 2005 [63]	Sweden	To elucidate the family's lived experience when a child in the family was diagnosed with cancer.	Cross‐sectional qualitative study (phenomenology)	*N* = 34: Mothers (*n* = 17), fathers (*n* = 12), patient (*n* = 5)	9–11 (median = 11)	Female: *N* = 1 Male: *N* = 4	Less than a month	Leukemia, brain tumor, solid tumor	Surgery, chemotherapy, radiation therapy	Newly diagnosed, active treatment	Interview	Data collection
Enskär & von Essen, 2000 [64]	Sweden	To describe what aspects of care are important for making children with cancer feel cared for, and to describe what kind of assistance, if any, these children need outside of the hospital.	Cross‐sectional qualitative study (descriptive)	*N* = 88: children (*n* = 25), parents (*n* = 31), nurses (*n* = 32)	8–12 (Mean = 9.6, SD = 1.5)	Female: *N* = 13 Male: *N* = 19^a^	1 month to over 5 years	Leukemia, lymphoma, Wilms' tumor, hepatoblastoma, osteogenic sarcoma, other solid tumors	Chemotherapy, radiation therapy, surgery, bone marrow transplantation	Active treatment, off treatment	Semi‐structured interview	Data collection
Hockenberry‐Eaton et al., 1999 [65]	United States	To define fatigue experienced by children with cancer and to begin development of a conceptual model.	Cross‐sectional qualitative study (descriptive)	Children only: *N* = 14	7–12 (Mean = 10)	Female: *N* = 6 Male: *N* = 8	1–95 months (Mean = 16)	Leukemia, lymphoma, solid tumor	Chemotherapy, radiation therapy, surgery	Active treatment	Focus group interview	Data collection, member checking
Enskär et al., 1997 [66]	Sweden	To identify children's experience of problems related to their cancer and the disease‐effect on the child's life situation.	Mixed‐methods descriptive study (exploratory design)	*N* = 10: children (*n* = 5), parents (*n* = 1)	6.5–12.5 (Mean = 8.5, SD = 2.3)	Female: *N* = 5 Male: *N* = 5	0.9–4.5 years (Mean = 2.6, SD = 1.8)	Leukemia, sarcoma	Chemotherapy, radiation therapy, surgery, bone marrow transplantation	Not specified (involving relapse)	Interview with drawing aid, a preliminary list of problems to measure disease‐ and treatment‐related problems in children or adolescents with cancer	Data collection
Ellerton & Turner, 1992 [67]	Canada	To examine the “Back to school” program's effects on classmates' cancer knowledge and attitudes, and explore children with cancer's concerns and coping behaviors when returning to school.	Mixed‐methods single‐armed study (convergent design)	*N* = 67: children with cancer (*n* = 3), classmates (*n* = 64)	7–10 (Mean = 8.7, SD = 1.5)	Female: *N* = 3	Not specified	Not specified	Not specified	Newly diagnosed	Semi‐structured interview, Children's coping strategies Inventory	Data collection
Mullis et al., 1992 [68]	United States	To investigate and compare the self‐esteem of school‐age children with leukemia to that of healthy children.	Cross‐sectional quantitative study (descriptive)	*N* = 63: children with leukemia (*n* = 13), healthy children (*n* = 50)	6–11 (Mean = 7.62)	Female: *N* = 7 Male: *N* = 6	Not specified	Acute lymphocytic leukemia, acute non‐lymphocytic leukemia	Chemotherapy (majority)	Not specified	Kinetic family drawing‐revised, Coopersmith self‐Esteem Inventory	Data collection
Munet‐Vilaró & Vessey, 1990 [69]	United States	To describe how Hispanic children explain the experience of having leukemia.	Cross‐sectional qualitative study (descriptive)	Children only: *N* = 23	6–12	Female: *N* = 8 Male: *N* = 15	Not specified	Acute lymphoblastic leukemia	Not specified	Active treatment	Semi‐structured interview	Data collection

^a^ Sex/gender data refer to all 32 children represented across child and parent interviews, not only the 25 children interviewed directly.

Twenty‐nine studies (74.4%) included only children with cancer, whereas the other 10 included one or more additional participant groups: parents (*n* = 7, 17.9%), nurses (*n* = 4, 10.3%), or peers without cancer (*n* = 3, 7.7%). Child sample sizes ranged from 1 to 78 (median = 15), with mean ages typically ranging from 8 to 10 years. Information on children's sex or gender was reported in 37 studies (94.9%), all using binary female/male categories; 34 studies (87.2%) included both female and male participants, while three (7.7%) included participants from only one category. Studies most often involved children receiving active treatment (*n* = 25, 64.1%), while four studies (10.3%) included newly diagnosed children, seven (17.9%) addressed post‐treatment stages, two (5.1%) addressed palliative stages, and eight (20.5%) included children who had experienced relapse. These categories were not mutually exclusive, and relapse‐specific findings were rarely reported separately. Leukemia (*n* = 33, 84.6%) and lymphoma (*n* = 19, 48.7%) were the most frequently reported diagnoses, and chemotherapy was the most frequently reported treatment (*n* = 18, 46.2%).

Qualitative designs predominated (*n* = 30, 76.9%), including 28 cross‐sectional studies (71.8%) and two longitudinal studies (5.1%). These comprised 22 descriptive studies (56.4%), five phenomenological studies (12.8%), one grounded theory study (2.6%), and two case‐based studies (5.1%). Three studies (7.7%) used quantitative designs, including two cross‐sectional studies (5.1%) and one prospective cohort study (2.6%). Six studies (15.4%) used mixed‐methods designs, four of which evaluated interventions (10.3%): two randomized controlled trials (5.1%), one single‐arm study (2.6%), and one feasibility and acceptability study (2.6%). Data were primarily collected through semistructured interviews (*n* = 26, 66.7%) and frequently incorporated creative methods such as drawing (*n* = 17, 43.6%), photography (*n* = 3, 7.7%), and puppetry (*n* = 1, 2.6%). Children most commonly participated as data contributors (*n* = 32, 82.1%), with limited use of member checking (*n* = 5, 12.8%) or participatory approaches (*n* = 3, 7.7%).

### 3.3 Meaning Making in School‐Aged Children With Cancer

Findings are organized into four interrelated domains derived from Park's meaning‐making model: global meaning (children's beliefs, developmental and healthcare‐related goals, and sense of meaning or purpose); appraised meaning and associated distress (the meanings assigned to cancer‐related experiences and why they are perceived as distressing); meaning‐making processes (the cognitive, emotional, socially informed, spiritual, and experiential ways children construct and process meaning, including limiting engagement with distressing information or experiences); and meanings made (identifiable changes over time in acceptance, causal understanding, perceived growth, identity, beliefs, goals, or sense of meaning). Table 4 presents the themes and subthemes within each domain, along with their definitions, illustrative quotations, and contributing studies.

**TABLE 4:** Summary of meaning‐making themes, definitions, and example quotes from contributing studies (*N* = 39).

Themes	Subthemes	Definitions	Example quotes	Involved studies
				*N* = 33
Global Meaning				
Beliefs (*n* = 17)	Optimistic beliefs about illness	Beliefs that cancer is temporary or curable and that children can recover, return to their pre‐cancer lives, and have a positive future.	“Happy that I knew what it [the diagnosis] was. No worry. No scared feelings. Mostly happiness. Really relieved hospitals look after you.” (age 8) [42, p. 9] “[I think…I'm going to get rid of it and it's going to go by and I'm young, so it's not like I am going to live with it for…the rest of my life. . .” (age 9) [57, p. 349]	*n* = 9: 31, 34, 41, 42, 50, 52, 57, 63, 69
	Beliefs about fairness and predictability	Beliefs about whether illness and treatment are fair, predictable, understandable, or controllable.	“[The child felt] a lot of sadness, because I thought I would never have it, this disease [cancer]. I Thought it was never going to happen to me.” (age 10) [44, p. 1324] “Why just I had to be sick.” [66, p. 23]	*n* = 4: 44, 61, 62, 66
	Beliefs about relationships	Beliefs about whether important relationships will remain stable, reciprocal, and accepting during illness.	“Someone who understands what you want and like…someone who, yes, someone who always supports you when you say something…” [51, p. 159] “I still have friends and they are still going to think that I am who I am.” (age 10) [57, p. 349]	*n* = 3: 51, 55, 57
	Beliefs about oneself	Beliefs about one's identity, difference from others, competence, and self‐worth in relation to cancer.	“Well, I will have to draw myself with hair that looks like a boy, since now I look like a boy … but my mom says it should grow back soon.” (age 8) [40, p. 234] “A girl without hands; a boy wearing a mask” (drawing description) [55, p. 114]	*n* = 3: 40, 55, 68
Developmental goals (intrapersonal aspects, *n* = 15)	Play and activity	The desire to continue enjoyable play, movement, and age‐appropriate activities.	“I wish we had fun games like sports and archery. …Basketball, castle, etc. I had these in my school.” (age 8) [33, p. 223] “The playroom is the only hope and joy for children. Without it, we will be dying.” (age 12) [38, p. 389]	*n* = 12: 31, 33, 36, 38, 45, 46, 48–50, 54, 61, 66
	Routine and normalcy	The goal of preserving familiar routines and a sense of ordinary life.	“I can't have ice cream now. But later, when I get better and get discharged, I can have ice cream” (age 8) [31, p. 69] “I am happy because I have a school in the hospital. I Also have a school outside.” (age 8) [33, p. 223]	*n* = 8: 31, 33, 34, 36, 38, 43, 54, 63
	Future‐oriented goals	Plans, hopes, and desired possibilities extending beyond the present illness experience.	“I also had this thought in my mind that I want to become a teacher.” (age 8) [31, p. 69] “I want to never have leukemia and never get sick again. I Want to go to school and want my hair big!” [43, p. 1147]	*n* = 3: 31, 43, 54
Developmental goals (relational aspects, *n* = 16)	Peer participation and sense of belonging	The goal of maintaining friendships, shared activities, and inclusion among peers.	“I think this place (the hospital classroom) positively affects communication with others (other children in the hospital).” (age 9) [33, p. 223] “The playroom makes me happy because I can see my friends, but it's not open every day. I Like to go there whenever I don't need to receive medication.” (age 6) [38, p. 389]	*n* = 13: 33, 36, 38, 40, 41, 46, 48, 51, 57, 60, 61, 63, 66
	Family togetherness and secure attachment	The desire for closeness, continuity, and emotional security with family members and pets.	“My father because he does everything to make me happy.” [48, p. 80] “The most important thing is that Mum and dad are with me all the time.” (age 9) [54, p. 466]	*n* = 8: 31, 36, 38, 48, 49, 54, 61, 66
Developmental goals (social aspects, *n* = 15)	School and competence	The goal of continuing education, learning, achievement, and age‐appropriate competence.	“I can catch up with my lessons thanks to the hospital classroom.” (age 12) [33, p. 9] “If you take your medication, you will get well soon, and you can go to school.” (age 8) [38, p. 389]	*n* = 14: 33, 36, 38, 40, 41, 51, 54, 57, 60–63, 66, 67
	General engagement and sense of connectedness	The desire to remain involved in and connected with life beyond the hospital.	“The computer really helps to keep me close to other people.” (age 12) [50, p. 147]	*n* = 1: 50
Healthcare goals (personal goals, *n* = 20)	Maintaining positive emotions and hope	The goal of sustaining enjoyment, reassurance, optimism, and hope for recovery.	“Oh, it's hard, you might cry. However, after a while now I have hope. My spirit should be cheerful.” (age 12) [31, p. 70] “In a way it (hearing the bell ring), it made me feel like I can get through this. I Can get through this and one day I will ring that bell good and gold.” [34, p. 5]	*n* = 10: 31, 33, 34, 46, 48, 49, 54, 63, 64, 66
	Participation in care	The desire to receive information, ask questions, express preferences, and take part in care decisions.	“I want to know what is going on, not just my parents know what's going on.” (age 9) [32, p. 131] “[The]Doctor…speaks secretly to my mom and dad from me. I Think they should talk beside me. I Think we should talk all together. After all, the patient is me.” (age 9) [37, p. 663]	*n* = 8: 32, 37, 39, 42, 47, 50, 63, 64
	Relief from suffering and achieving recovery	The goal of reducing symptoms and treatment burdens and becoming well.	“The doctor also said, ‘There's nothing wrong with you; you're fine.’ this means hope.” (age 9) [31, p. 71] “I wanted to get rid of the line so I could do stuff I wasn't able to do.” [34, p. 7]	*n* = 8: 31, 32, 34, 36, 38, 42, 46, 50
	Interpersonal support for coping and recovery	The goal of having companionship and interpersonal support to help cope with cancer and treatment, relieve distress, and promote recovery.	“I get company [to help myself feel better about cancer and treatment].” (age 11) [52, p. 8] “[I] try and make as many people come visit me as I can.” (age 11) [57, p. 349]	*n* = 4: 52, 57, 63, 64
Healthcare goals (provider‐related expectations, *n* = 14)	Emotional responsiveness	Expectations that providers recognize, validate, and respond sensitively to children's emotions.	“She said that while I was losing my hair, we could keep it to make a nest for the owl babies. It made me feel better about losing my hair.” (age 11) [32, p. 131] “I love my nurse here; she measures my blood pressure and asks ‘how are you dear…’ before measuring.” (age 7) [37, p. 662]	*n* = 9: 32, 37–39, 43, 47, 60, 63, 64
	Competent and developmentally responsive care	Expectations for skilled and timely care that relieves discomfort and responds to children's practical and developmental needs.	“I feel very safe with him because once I had an allergic reaction and he had to stick me with the needle thing and I was able to start breathing again.” (age 12) [32, p. 132] “I love my nurse Mrs.. She does not hurt when she takes blood, she finds it in 2 minutes. I Do not get hurt because she gets it right away.” [37, p. 663]	*n* = 9: 32, 33, 37, 39, 43, 47, 60, 63, 64
	Effective communication	Expectations for clear, honest, developmentally appropriate explanations and opportunities to ask questions.	“When the nurses tell me what is going on and when everything is going to happen I felt less scared.” (age 9) [32, p. 131] “I knew what was going to happen so that was reassuring. The doctor was really good at telling me everything so I felt ok.” (age 8) [42, p. 10]	*n* = 8: 32, 37, 39, 42, 47, 50, 60, 61
	Playful interaction	Preferences for providers to use play, humor, and enjoyable engagement during care.	“His stories were funny and everything so it made us smile a lot and forget I was in the hospital.” (age 12) [32, p. 131] “Mrs… gives me a sense of hope. Because sometimes she plays with me, for example, when she comes to my room, she looks at my dolls and toys, and we play dolls together, or sometimes we paint together.” (age 6) [38, p. 389]	*n* = 8: 32, 37–39, 43, 50, 60, 64
	Respectful interaction	Expectations that providers treat children and families genuinely, courteously, and with respect.	“The doctor who listens to his patient is a good doctor. When he talks to him, he looks at his face.” (age 9) [37, p. 662] “There are nurses who are kind and listen to me. For example, when they inject the medicine, I tell them to inject it slowly, so I experience less pain. And they listen to me.” (age 7) [38, p. 389]	*n* = 6: 37, 38, 47, 54, 63, 64
	Warm and approachable demeanor	Preferences for providers whose personal qualities, appearance, and manner convey kindness, warmth, safety, and approachability.	“When they are wearing scrubs, I like pictures on it because it shows you a little about them.” (age 12) [32, p. 132] “They would always smile. Always! they were never sad. They never frowned. They were just always happy…that made me feel welcomed and happy to be here.” [39, p. 20]	*n* = 5: 32, 37–39, 47
Healthcare goals (caregiver‐related expectations, *n* = 8)	Support in understanding and decision‐making	Expectations that parents help explain illness and treatment and support children's participation in decisions.	“I asked mum lots of questions. Sometimes I don't like to ask questions to staff. (I) usually just ask mum afterward.” (age 8) [42, p. 12] “The doctors talk to me, but when I don't understand, my mother explains it.” (age 12) [50, p. 146]	*n* = 5: 42, 45, 46, 50, 54
	Emotional comfort and reassurance	Expectations that parents provide security, encouragement, and relief from fear or distress.	“Hope means mommy because mommy is nice and plays with me in the hospital.” (age 9) [31, p. 69] “It really helped having my mum, my dad and my mum's best friend with me…my mum was there to reassure me.” (age 11) [42, p. 12]	*n* = 4: 31, 42, 46, 54
	Instrumental caregiving support	Expectations that parents provide practical help with daily needs, symptoms, and treatment.	“[Parents] overseeing oral medication administration.” (drawing description) [46, p. 2732] “[To deal with treatment, I] get my mom to massage me.” (age 11) [52, p. 7]	*n* = 3: 46, 52, 60
Appraised Meaning and associated distress				*N* = 31
Threats to the self (*n* = 26)	Entrapment in ongoing treatment and suffering	Appraisals of illness and treatment as persistent, inescapable, painful, and beyond one's control.	“It's a bad experience, which seems to have no end, is a lot of suffering.” (age 7) [44, p. 1324] “I felt deep pain. I can't explain it. I don't feel it anymore. I Cried and cried because I wanted it to go away.” [53, p. 395]	*n* = 15: 34, 36, 38, 44–47, 50, 53, 54, 57, 58, 60, 66, 69
	Loss of agency and control	Appraisals of being denied information, opportunities to ask questions, or meaningful involvement in care.	“(I would be) doomed if I ask anymore…I wouldn't be allowed to know…no idea why they won't let me know.” (age 8) [42, p. 11] “The doctor and nurse always provided detailed information about lumbar puncture to my parents, but not me. I Think I have a right to know about it because I am the one who underwent lumbar puncture.” [47, p. 3332]	*n* = 9: 32, 34, 37, 39, 42, 43, 47, 61, 62
	Concern about visible bodily changes	Appraisals of appearance changes as threats to self‐image, social acceptance, or identity.	“The hair? [She runs her hand through the hair]. It fell all over…I Got depressed. I Was ugly.” [43, p. 1144] “I wasn't sure what was going to happen, and I was really afraid of my hair falling out.” (age 10) [57, p. 348]	*n* = 8: 37, 40, 43, 50, 55, 57, 63, 67
Threats to normalcy (*n* = 18)	Hospitalization and disruption of everyday life	Appraisals of cancer, hospitalization, and treatment as separating children from ordinary life and disrupting familiar routines, roles, and developmental experiences.	“I do not like it here… Too bad… Today is my birthday; I want to eat chocolate cake. The time never goes by in here.” [43, p. 1146] “I do not like waking up early to weigh…” [43, p. 1145]	*n* = 10: 31, 36, 38, 43, 49, 53, 57, 60, 65, 66
	Separation from family, peers, and school	Distress arising when illness‐related separation disrupts children's usual relational routines and participation in family, peer, and school life.	“I was starting my school life, I wanted to learn to read and write, I started to write my name […] I have a lot of friends at school and my teacher was cool.” (age 6) [41, p. 8] “[The hard part of finding out I had cancer was] that I had to stay up here and I barely got to see my family.” (age 10) [57, p. 348]	*n* = 9: 33, 40, 41, 44–46, 53, 57, 66
	Restricted participation in normal activities	Appraisals of illness and treatment as limiting participation in play, movement, and other usual or valued activities.	“I stopped going there, because I started my treatment, and I cannot take a river bath, I cannot do anything anymore.” (age 8) [41, p. 8] “It stops me. It is always slowing me down. I can't go to the park…There are so many things I'm not allowed…There is so much I can't do.” (age 8) [42, p. 9]	*n* = 5: 34, 41, 42, 45, 66
Threats to relationships (*n* = 6)	Worry about impact on family and others	Concern that illness may distress, burden, separate, or harm important others.	“I was also thinking about my family and how they were going to manage…I thought it would be hard on them as well.” (age 11) [42, p. 12] “She cried a lot. I Said “cry not mom… It does not hurt…”. She always cries… I Feel sad when she cries because she is sad too. Sometimes I stay and I do not cry… Then my mom is happy.” [43, p. 1145]	*n* = 5: 42–44, 47, 63
	Grief and loss	Meaning assigned to actual or anticipated losses associated with illness, relationships, or death.	“This girl is sad because she is separated from her sister and is grieving a friend who died of leukemia.” (drawing description; age 7) [45, p. 296]	*n* = 1: 45
Threats to the future (*n* = 17)	Uncertainty	Distress arising from unclear illness, treatment, relapse, and future outcomes.	“I'm afraid of it [the tumor], I'm really scared that it will come back, right? That it will turn into something much worse.” (age 12) [50, p. 147] “[Finding out I had cancer] was just really, really scary for me…I kind of didn't know what was going to happen next.” (age 9) [57, p. 347]	*n* = 13: 32, 34, 42, 44, 46, 47, 50, 55, 57, 58, 63, 66, 69
	Threat of death	Appraisals of cancer as life‐threatening, including fears of death and concerns about dying in pain, alone, or separated from loved ones.	“I already thought bad things, things of death. Because ‘everyone’ dies [referring to occurrences of the death of other patients].” [43, p. 1144] “I was afraid to die, and not having my mother and father next to me, it is difficult […].” (age 7) [44, p. 1324]	*n* = 8: 41, 43, 44, 47, 54, 57, 58, 61
Meaning‐making processes				*N* = 31
Meaning‐focused coping (*n* = 23)	Searching for comprehensibility	Efforts to understand illness and treatment by seeking and integrating information about relevant concepts, causes, consequences, and possible solutions.	“It is leukemia. That is what we call it. But you can treat this now.” (age 11) [42, p. 11] “That was my disease, I felt pain in my legs and arms, I usually had to lie down.” [53, p. 395]	*n* = 10: 40–42, 45, 52, 53, 59, 63, 65, 67
	Positive reappraisal	Efforts to reinterpret an illness experience by identifying positive aspects, benefits, personal growth, or valued changes that may arise from it.	“I think that death is a good thing, since after that, my colleagues went to a better place, where they do not feel any more pain and no longer need to undergo QT sessions, they are completely free.” (age 11) [41, p. 8] “But sometimes it's nice [oncology unit]. There are nice people here.” [43, p. 1146]	*n* = 8: 32–34, 39, 41, 43, 60, 66
	Searching for significance	Efforts to understand why illness‐related experiences matter and how they relate to recovery, survival, or broader life concerns.	“I do the CT [chemotherapy] because I want to get well and go to my house.” [43, p. 1147] “I accept it because I know this treatment serves to kill the disease.” (age 10) [50, p. 146]	*n* = 7: 42, 43, 50, 53, 54, 62, 66
	Visual metaphors^a^	Using concrete images or symbols to represent emotions, illness experiences, or abstract meanings.	“Mountains represent hope because they are always high and strong.” (age 11) [31, p. 70] “Sick days: Storm with a sad face and tears.” (age 10) [46, p. 2735]	*n* = 6: 31, 41, 42, 45, 46, 54
	Selective appraisal	Efforts to reduce the perceived severity of an illness experience by comparing it with worse possible or previously experienced circumstances.	“Whenever I compare my current status with the first month of my cancer diagnosis and hospitalization, I feel better.” (age 8) [38, p. 389]	*n* = 2: 38, 63
	Illness identity negotiation	Efforts to determine how cancer experiences and illness‐related changes or symbols fit within one's developing sense of self.	“I put it [a necklace received from a nurse for the end of treatment] on, then I took it off, then I lost it, then I found it, then I put it on again, then off again, then lost it, then found it. And then I thought you know what, I am going to keep it on for the rest of my life.” [34, p. 6]	*n* = 1: 34
Emotion‐based meaning processing (*n* = 12)	Emotional processing	Efforts to identify, experience, and reflect on emotions to understand what and why one is feeling and derive meaning from illness‐related experiences.	“I can feel sad when I am ill. I Can also get stressed. The hospital classroom is a place here that makes me feel happy.” (age 9) [33, p. 223] “I am happy because everything is working out…I am getting better.” (age 11) [42, p. 12]	*n* = 12: 32, 33, 38, 39, 42, 46, 48, 52, 55–57, 65
Socially informed meaning making (*n* = 11)	Interpreting social and symbolic cues^a^	Interpreting illness and care through others' verbal or nonverbal behavior and socially shared symbols.	“I knew that it’s bad ’cos of her (mother's) reactions and the way she is.” (age 11) [42, p. 12] “When lumbar puncture report was ready, a doctor called my mother. Her eyes were red and swollen when she came back, but she said that everything was okay. However, I still did not feel good (shedding tears and sobbing)” [47, p. 3331]	*n* = 10: 32, 34, 37–39, 42, 43, 45–47
	Meaning making through others' guidance^a^	Using explanations, reassurance, or direction from trusted others to understand illness.	“I was very sorry at first but then the nurse here said to me by smiling that my hair will come out when I beat this disease. I Was happy.” (age 12) [37, p. 661] “I took a picture of my mother because my mother says, if you take your medication, you will get well soon, and you can go to school.” (age 8) [38, p. 389]	*n* = 4: 32, 37, 38, 40
	Meaning making through similarity and vicarious experience^a^	Developing connection through perceived similarities with others and forming hope or positive expectations from observing others' cancer experiences and treatment trajectories.	“It's like everybody, everybody, people go through treatment and everybody finish, then those people have to finish it. Like you can't just continue going through treatment and never end.” [34, p. 5] “It is nice to talk to a nurse who has things in common with me.” [39, p. 21]	*n* = 2: 34, 39
Avoidance‐related meaning processing (*n* = 11)	Distancing	Disengaging from threatening illness‐related information, thoughts, or experiences to limit distress.	“I just blocked out completely because I didn't want to hear it anymore.” (age 12) [42, p. 9] “I just don't really think about [my cancer]… when somebody tries to help me, I push them off cause I don't want to think about everything.” (age 12) [52, p. 7]	*n* = 10: 34, 35, 42, 50, 52, 56–58, 62, 67
	Label avoidance^a^	Avoiding the word or identity of cancer to reduce threat, stigma, or emotional discomfort.	“Then, she saw I had the fever and early in the morning we went to the hospital. Then, they found out this thing.” [53, p. 394]	*n* = 1: 53
	Self‐silencing^a^	Refraining from asking questions or expressing illness‐related knowledge, thoughts, or emotions when children feel that doing so is not permitted or welcomed.	“Yeah, whatever they (doctors) asked me, I'd answer. But I would not like, say anything that isn't, that they didn't, if they didn't have a question, but I had an answer, I would just keep that until they asked that question.” [34, p. 7]	*n* = 1: 34
Spiritual meaning making (*n* = 5)	Religious meaning making	Drawing on faith, prayer, or connection with a higher power to interpret illness and sustain hope, comfort, and strength.	“This is the praying room, here mothers pray for our health. It gives me a sense of hope that we will get better.” (age 10) [38, p. 389] “To finish this treatment, I went to church, prayed and thank god everything is right!” (age 12) [50, p. 147]	*n* = 4: 31, 38, 50, 52
	Afterlife meaning making	Drawing on beliefs about the afterlife to reinterpret death as freedom from illness, treatment, and suffering.	“Because I will not have these things from here, having to go to the hospital, to get sick. Everything will be different there [in the afterlife].” (age 6) [41, p. 8]	*n* = 1: 41
Experiential meaning making (*n* = 5)	Sensory and embodied interpretations^a^	Constructing meaning through direct sensory and bodily experiences of illness and care environments.	“The playing room is colorful; the pictures of beautiful animals are painted on the walls. There are a lot of toys in there, which are not available outside of the playing room.” (age 6) [38, p. 389] “I like flowers and plants… nice colors and foliage… Many flowers are colourful and you feel good about that.” [48, p. 80]	*n* = 4: 38, 42, 48, 58
	Schema‐based interpretations^a^	Interpreting unfamiliar illness or treatment experiences through existing knowledge, language, memories, and expectations.	“I was so nervous when I heard the word ‘lumbar puncture’. It must be very painful because it is ‘a puncture.’’’ [47, p. 3331]	*n* = 1: 47
Meanings made				*N* = 1
Acceptance (*n* = 1)		An achieved recognition of the reality and necessity of illness or treatment.	“I had to deal with things…I have to accept the treatment because I would not be able to make it without it.” (age 11) [42, p. 11]	*n* = 1: 42
Changed beliefs (*n* = 1)		Identifiable shifts in beliefs about illness, recovery, the self, or the future resulting from meaning‐making efforts.	“It's curable. Soon I will be having a body like everyone else.” (age 11) [42, p. 11]	*n* = 1: 42

^a^ Inductively identified child‐specific meaning‐making processes not explicitly represented in Park's meaning‐making model.

### 3.4 Global Meaning

Evidence of children's global meaning was identified across 33 studies (84.6%) and encompassed broader beliefs and developmental and healthcare‐related goals. In nine studies (23.1%), children held optimistic beliefs that cancer was temporary and curable, expecting full recovery, a return to pre‐illness life, and a positive future, even when such outcomes were clinically uncertain or unlikely. In four studies (10.3%), limited control over cancer and treatment appeared to prompt children to ask “why me,” view having to undergo unwanted procedures as unfair, and express disbelief that cancer had happened to them, reflecting beliefs about life's fairness and predictability. Five studies (12.8%) also addressed children's beliefs about relationships and the self. Relational beliefs (*n* = 3, 7.7%) emphasized supportive, reciprocal friendships and the expectation of being accepted and seen no differently despite cancer. Beliefs about oneself (*n* = 3, 7.7%) reflected children's views of their identity, difference from others, competence, and self‐worth in relation to cancer; some perceived bodily changes as setting them apart from peers without cancer or diminishing their self‐worth. One study contributed to both subthemes.

Children's developmental goals reflected what they valued during illness and encompassed intrapersonal (*n* = 15, 38.5%), relational (*n* = 16, 41.0%), and social aspects (*n* = 15, 38.5%). Intrapersonal goals included play and age‐appropriate activities, familiar routines and pleasures that preserved or restored normalcy, and plans and aspirations extending beyond the present illness. Relational goals centered on peer participation and belonging through friendships and shared activities and on family togetherness and security through closeness with parents, siblings, and pets. Social goals reflected children's desire to continue learning, keep pace with schoolwork, maintain competence, and return to school following recovery, whereas one study highlighted internet use as a means of remaining connected to life beyond the hospital.

Within children's global meaning systems, developmental priorities intersected with the demands of cancer and treatment, shaping healthcare‐related goals (*n* = 20, 51.3%). These included maintaining positive emotions and hope (*n* = 10, 25.6%), participating in care (*n* = 8, 20.5%), seeking relief from suffering and recovery (*n* = 8, 20.5%), and obtaining interpersonal support for coping and recovery (*n* = 4, 10.3%). Specifically, children sought reassurance about recovery and treatment completion, clear information and opportunities to ask questions and express preferences, relief from symptoms and treatment burdens, and companionship that eased distress and supported recovery.

Provider‐related expectations (*n* = 14, 35.9%) emphasized emotional responsiveness (*n* = 9, 23.1%), competent and developmentally responsive care (*n* = 9, 23.1%), effective communication (*n* = 8, 20.5%), playful interaction (*n* = 8, 20.5%), respectful interaction (*n* = 6, 15.4%), and a warm and approachable demeanor (*n* = 5, 12.8%). Children preferred providers who responded to their emotions, fostered a positive environment, communicated clearly, invited questions, and provided timely, skillful, and creative care that relieved discomfort and met developmental needs. They also valued providers who incorporated play, interacted genuinely and respectfully with children and families, and conveyed warmth, kindness, and approachability. Caregiver‐related expectations (*n* = 8, 20.5%) included support in understanding and decision making (*n* = 5, 12.8%), emotional comfort and reassurance (*n* = 4, 10.3%), and instrumental caregiving support (*n* = 3, 7.7%). Children expected caregivers to interpret and clarify information, particularly when they preferred not to receive it directly or had difficulty understanding providers, and to support their participation in decisions. They also expected reassurance and practical assistance with daily needs, symptoms, and treatment. No studies addressed children's subjective sense of meaning or purpose.

### 3.5 Appraised Meaning and Associated Distress

Across 31 studies (79.5%), appraised event meaning and associated distress reflected how children interpreted cancer‐related experiences as threats to the self (*n* = 26, 66.7%), normalcy (*n* = 18, 46.2%), relationships (*n* = 6, 15.4%), and the future (*n* = 17, 43.6%). Within threats to the self, entrapment in ongoing treatment and suffering (*n* = 15, 38.5%) captured perceptions of repeated pain, procedures, and treatment demands as persistent, uncontrollable, and inescapable, with no clear end or prospect of relief. Loss of agency and control (*n* = 9, 23.1%) arose when adults withheld information, dismissed children's questions or preferences, excluded them from care discussions, or avoided conversations about diagnosis, treatment, and death, contributing to confusion, frustration, and powerlessness. Concerns about visible bodily changes (*n* = 8, 20.5%), particularly hair loss, centered on threats to self‐image, identity, and social acceptance, including fears of looking different or being bullied [43, 67].

Cancer was also appraised as a profound disruption to normalcy (*n* = 18, 46.2%). Hospitalization and treatment disrupted everyday life (*n* = 10, 25.6%) and, for some children, came to symbolize isolation and despair [31]. Illness‐related separation from family, peers, and school (*n* = 9, 23.1%) interrupted familiar relational routines, schooling, developmental milestones, and sources of belonging, while treatment restrictions limited participation in play, movement, and other valued activities (*n* = 5, 12.8%), producing a sense of deprivation and discontinuity. Threats to relationships (*n* = 6, 15.4%) arose from concerns about the effects of illness on family members and others (*n* = 5, 12.8%), including fears of burdening or being separated from them, siblings developing cancer, or the family breaking down following the child's death. Children also experienced grief over actual or anticipated losses (*n* = 1, 2.6%). Threats to the future (*n* = 17, 43.6%) centered on uncertainty about the disease, treatment, relapse, and future outcomes (*n* = 13, 33.3%), including fears of worsening illness, prolonged suffering, recurrence, or treatment failure. Children also appraised cancer as life threatening (*n* = 8, 20.5%), encompassing not only fear that they might die but also concerns about how death might occur, particularly dying in pain or separated from loved ones.

### 3.6 Meaning‐Making Processes

Meaning‐making processes encompassed the automatic or deliberate ways school‐aged children sought to reduce discrepancies between global and appraised event meaning. Across 31 studies (79.5%), children's meaning making involved cognitive, emotional, socially informed, spiritual, experiential, and avoidance‐related processes that could facilitate understanding and reinterpretation, situate cancer within their relationships, spiritual beliefs, embodied experiences, and developing identities, or regulate their engagement with distressing illness‐related information, thoughts, labels, and emotions.

Meaning‐focused coping (*n* = 23, 59.0%) encompassed children's deliberate efforts to understand, evaluate, or revise illness‐related meanings. Children searched for comprehensibility (*n* = 10, 25.6%) by seeking, interpreting, and integrating information about illness and treatment into their existing understanding. Examples included describing symptoms using medical and child‐specific language in terms of their causes, management, and consequences [59], and comparing the afterlife to familiar experiences [41]. Positive reappraisal (*n* = 8, 20.5%) involved efforts to identify positive aspects, benefits, or valued changes arising from illness, including developing emotional bonds with nurses, continuing education in flexible and playful ways, viewing treatment completion as an achievement, appreciating increased attention from others, and reframing death as freedom from suffering [33, 34, 39, 41, 66]. Children searched for significance (*n* = 7, 17.9%) by understanding medical examinations, treatment, cooperation with providers, hospitalization, and a healthy diet as necessary to feel better, recover, and avoid death. Treatment was often understood as their “only choice,” as reflected in the statement, “*Leukemia only goes away with chemo.*” [43, p. 1141; 50],

Children also used visual metaphors (*n* = 6, 15.4%) to represent emotions, understandings of illness, and abstract concepts such as hope and death, translating them into concrete, familiar images that facilitated understanding and meaning reconstruction. In a longitudinal case study, a child depicted cancer as a “dangerous dragon” and chemotherapy as “little soldiers.” As the illness progressed to the palliative stage, the dragon grew larger and darker while the soldiers disappeared, visually conveying a shift from cancer as potentially defeatable to cancer as ultimately prevailing [54]. Selective appraisal (*n* = 2, 5.1%) reduced the perceived severity of illness by comparing the present situation with potentially worse or previously experienced circumstances, such as improvement relative to the first month following diagnosis and hospitalization [38]. One study (2.6%) illustrated illness identity negotiation through a child's ambivalent engagement with a necklace symbolizing treatment completion. Repeatedly wearing and removing it before deciding to keep it reflected negotiation over whether and how the cancer experience became part of the child's developing identity [34].

Emotion‐based meaning processing (*n* = 12, 30.8%) involved identifying, experiencing, and reflecting on emotions to understand what children were feeling and why and to derive meaning from illness‐related experiences. Children used drawing, journaling, and verbal reflection to connect emotions with particular experiences and distinguish “good” from “sick” days based on their emotional responses [46, 55, 57]. Socially informed meaning making (*n* = 11, 28.2%) involved constructing meaning through interactions with others and information obtained from them. Children interpreted social and symbolic cues (*n* = 10, 25.6%), such as providers' appearance and behavior, parents' facial expressions, and the treatment‐completion bell, to infer approachability, illness severity, achievement, or closure [34, 37, 47]. They also drew on explanations and reassurance from trusted others (*n* = 4, 10.3%). Perceived commonalities fostered connection, while observing others who had completed treatment provided role models and hope for their own recovery (*n* = 2, 5.1%).

Avoidance‐related meaning processing (*n* = 11, 28.2%) involved disengaging from threatening illness‐related information, thoughts, or experiences to limit distress. Distancing (*n* = 10, 25.6%) included blocking out information, avoiding thoughts about cancer, and resisting others' attempts to discuss the illness or offer help. Label avoidance (*n* = 1, 2.6%) was illustrated by a child referring to cancer as “this thing.” Such indirect language may have reduced perceived threat, stigma, or emotional discomfort while also reflecting adults' health literacy and attitudes toward cancer and the broader sociocultural meanings attached to it, which shaped how children interpreted and discussed their illness [53]. Self‐silencing (*n* = 1, 2.6%) occurred when children refrained from asking questions or expressing illness‐related knowledge, thoughts, or emotions because doing so did not seem permitted or welcomed. Rather than directly reframing illness, these processes limited engagement with threatening meanings and showed how children's meaning processing could be both protective and shaped by what adults and sociocultural contexts communicated or left unspoken.

Spiritual meaning making (*n* = 5, 12.8%) included drawing on religious faith, prayer, or connection with a higher power to interpret illness and sustain hope, comfort, and strength (*n* = 4, 10.3%). One study (2.6%) described afterlife meaning making, in which death was reinterpreted as entry into a different existence free from illness, hospitalization, treatment, and suffering. Experiential meaning making (*n* = 5, 12.8%) involved children constructing meaning through direct encounters with their bodies and care environments. Sensory and embodied interpretations (*n* = 4, 10.3%) arose as children assigned meaning to bodily sensations and environmental features, shaping whether these experiences were understood as comforting, hopeful, or distressing. Schema‐based interpretations (*n* = 1, 2.6%) occurred when children interpreted unfamiliar treatments through existing language, knowledge, and expectations. For example, one child anticipated pain from a lumbar puncture because the familiar meaning of the word “puncture” implied that it must be painful [48].

### 3.7 Meanings Made

Evidence of meanings made was identified in one longitudinal study (*n* = 1, 2.6%), which documented changes over time in children's understanding, including greater acceptance of treatment and more positive beliefs about curability and recovery. These findings suggested that ongoing illness and treatment experiences could produce identifiable shifts in both appraised meaning and broader beliefs, although evidence linking these changes to psychosocial adjustment remained limited.

## 4 Discussion

This review provides the first comprehensive synthesis of meaning making among school‐aged children with cancer and supplements Park's [23] model by demonstrating that meaning making in this population is developmentally structured, relationally embedded, and socioculturally shaped. Children's global meaning systems encompassed beliefs and developmental and healthcare‐related goals concerning normalcy, belonging, participation, recovery, supportive care, and the future, together defining what mattered to them during illness. Cancer appraisals are shaped by children's developmental stage, physical and emotional states, access to information and shared decision‐making opportunities, and relational and sociocultural contexts.

Distress may arise when these appraised meanings conflict with children's global beliefs and goals, resulting in perceived threats to the self, normalcy, relationships, and future. Across the included studies, children negotiated cancer appraisals through cognitive, emotional, socially informed, spiritual, experiential, and avoidance‐related meaning‐making processes that facilitated developmentally congruent understanding and reinterpretation, supported perceived control and emotion regulation, or created distance from distressing meanings. These processes were relational and context‐dependent, shaped by interactions with caregivers, providers, and others, as well as by sociocultural, religious, and spiritual frameworks communicated through child–adult interactions. These findings highlight meaning making as a dynamic, developmentally grounded process rather than a simple stimulus‐response pattern. Finally, we found limited evidence regarding children's subjective sense of meaning or purpose and meanings made, likely reflecting methodological constraints rather than children's limited capacity to experience or express them.

Although developmental stage, disease course, and sociocultural context were not the primary focus of the included studies, the synthesis revealed several noteworthy patterns. Children's meaning making ranged from concrete, experience‐based interpretations expressed through play, drawings, and sensory or relational interactions to more abstract reflections on fairness, uncertainty, identity, and the future. Rather than representing a simple developmental progression, this variation likely reflects both the diversity of developmental capacities within middle childhood and differences in how children's perspectives were elicited. Studies using developmentally appropriate methods, such as drawing, photography, and storytelling, showed that school‐aged children could express complex reflections on hope, identity, future aspirations, and death. These findings suggest that children's capacity for meaning making may be underestimated when opportunities for developmentally appropriate expression are limited.

The included studies involved children at different points along the cancer trajectory, and several priorities recurred across the literature. Children sought to maintain normalcy through play, school participation, daily routines, and meaningful relationships while also seeking to understand their illness, participate in care, and have their perspectives acknowledged by parents and healthcare professionals. Although stage‐specific findings were rarely reported separately, these recurring priorities suggest that meaning making is not confined to a particular point along the illness trajectory but may represent an ongoing developmental process through which children seek to preserve normalcy, agency, and connection while adapting to changing illness experiences.

Synthesizing studies conducted across diverse cultural settings also revealed variation in the aspects of meaning making emphasized. Studies from Europe and North America appeared to focus more often on children's participation in care, communication with healthcare professionals, and developing autonomy. Studies from Brazil more commonly explored existential concerns surrounding suffering, death, and spirituality, whereas studies from parts of Asia more often highlighted hope, family relationships, and children's efforts to understand illness. Whether these patterns reflect sociocultural influences, differences in healthcare systems, or variations in study focus remains unclear and warrants further investigation.

Meaning making in school‐aged children is grounded developmentally in emerging coping efficacy and cognitive, emotional, and regulatory capacities that are shaped through manageable stressors, sufficient personal and interpersonal resources, and adaptive adult guidance [70]. These capacities support increasingly complex illness understanding and meaning construction while promoting resilience in the context of stress and adversity [71]. Although children may enter hospitalization with existing coping skills, illness treatment and hospital environments often require them to cope with “the unfamiliar” in the physical, emotional, and relational realms [72], while exclusion from treatment‐related decision making may contribute to feelings of insecurity and inferiority.

Developmental stage and lived experience jointly shape children's meaning making, including concrete, experience‐based sense making that connects illness with everyday life and familiar contexts [42, 72]. This process may support psychosocial adjustment and posttraumatic growth among pediatric cancer survivors [73]. However, existing research rarely examines developmental variation in meaning‐making processes, limiting our understanding of how meaning‐making capacities evolve across childhood [70]. Emerging evidence further suggests that meaning in life is both present and measurable among children aged 6–12 and may be cultivated through developmentally appropriate interventions [74]. Despite these advances, pediatric oncology research still lacks developmentally appropriate, child‐centered approaches to assessing meaning and purpose within patient‐reported outcomes, contributing to continued reliance on adult proxy reports.

## 5 Clinical Implications

These findings have important implications for psychosocial care in pediatric oncology. Supporting children's meaning making requires developmentally appropriate, relationship‐based, and child‐centered approaches that extend across the illness trajectory. Children with cancer make meaning within relational systems that include families, healthcare professionals, and broader social networks, which contribute to illness interpretation and identity reconciliation [75, 76, 77]. Families serve as the filter and primary milieu through which children interpret illness, highlighting the need for family‐centered communication interventions [78]. Logistically, this may include developmentally tailored medical and psychosocial resources for families, as well as peer or group‐based support to help caregivers engage in ongoing meaning making while navigating difficult illness‐related conversations and experiences [79, 80], including discussions surrounding disease progression or end of life.

Findings from this review also suggest that children rely on healthcare professionals to make sense of their illness, reduce uncertainty, and maintain a sense of normalcy through their approachable, developmentally appropriate communication. Care should therefore be aligned with what matters to children by incorporating their developmental priorities and healthcare expectations into goal‐concordant decision making. In partnership with parents, healthcare professionals can scaffold children's skills and confidence in care participation and self‐advocacy through medical play, communication tools, validation of feelings, and medical information provided in developmentally accessible formats [81, 82].

For children with serious illnesses, the process of meaning making carries important clinical implications for their psychological adjustment and overall quality of life [83]. Interventions to facilitate meaning making may be formal or informal and can be integrated throughout serious illness communication and pediatric palliative care, particularly during key care transitions, when children's developmental priorities and healthcare expectations may shift. Although children's needs for understanding, participation, normalcy, agency, and connection persist across the cancer trajectory, the emphasis of meaning‐making conversations may shift over time. Conversations may address illness understanding and immediate concerns at diagnosis; coping with treatment while maintaining routines and relationships during active treatment; identity, reintegration, future goals, and recurrence concerns during survivorship; and changing hopes, comfort, relationships, spirituality, or death during disease progression and palliative care, guided by the child's readiness.

Formal interdisciplinary interventions may support meaning making through positive reappraisal; narrative approaches, such as written or digital storytelling; arts‐based approaches, such as visual art, photography, and music; and legacy‐oriented activities [74, 84, 85]. Psychologists and social workers may explore illness appraisals, distress, and coping; child‐life specialists and art or music therapists may facilitate verbal and nonverbal expression; palliative care clinicians may connect children's priorities with goals of care; and chaplains may address existential, spiritual, or religious concerns. Meaning‐making conversations should be culturally responsive, as beliefs about autonomy, family roles, hope, suffering, death, and spirituality may shape how children understand and communicate about illness. Clinicians should explore these perspectives with each child and family rather than make assumptions based on cultural background. Interventions for school‐aged children should use more concrete language than those designed for adolescents and young adults, focus on one idea at a time, and provide developmentally appropriate modes of expression.

Informal bedside support can provide opportunities to explore evolving identity, illness integration, and personal narratives. In the absence of validated meaning‐focused assessments for this population, clinicians may open these conversations with brief questions such as, “What is most important to you right now?” “What do you think is happening, and what remains confusing?” “What helps you make sense of hard days?” or “Has cancer changed how you see yourself or your future?” Inviting children to share “their story” may provide insight into how illness is understood, positioned, or negotiated within their developing sense of self. When illness is not explicitly discussed within that narrative, responding with curiosity about how children relate illness to their broader life experiences and developmental priorities may create opportunities to support meaning making and identity integration. Together, these interventions may promote children's sense of autonomy and control while supporting adaptive coping and psychosocial adjustment. Meaning making should therefore be understood not simply as a coping response, but as a developmental process that can be supported and guided through clinical care.

## 6 Limitations

This study has several limitations. First, the search strategy and conceptual framing were informed primarily by Park's [23] meaning‐making model, which was largely developed based on adult populations and may not fully capture developmentally specific manifestations of meaning making in children. The proximity‐based search strategy may have missed relevant studies, while restricting inclusion to English‐language publications may have limited culturally diverse perspectives on meaning making and illness experiences. Moreover, findings were rarely disaggregated by age, sex/gender, time since diagnosis, cancer type, treatment type, or disease course. Most studies combined children across the school‐age years and reported demographic and clinical characteristics descriptively rather than examining how meaning‐making experiences varied according to these characteristics. This limited our ability to identify developmental, sex‐ or gender‐related, and illness‐related patterns. Future research should directly examine variation in children's meaning making across demographic, developmental, and clinical characteristics rather than treating school‐aged children as a homogeneous group and should employ longitudinal designs to better understand how meaning‐making processes evolve across the cancer trajectory. Greater attention is also needed to the development and evaluation of developmentally appropriate, child‐centered methods and patient‐reported measures that capture children's subjective experiences of meaning, purpose, and meanings made rather than relying solely on parent proxy reports. Finally, future studies using comparable methodologies across diverse cultural settings are needed to better understand how sociocultural contexts shape children's meaning‐making experiences.

## 7 Conclusion

In this review, we have highlighted meaning making as a dynamic, developmentally grounded, and relational process among school‐aged children with cancer. Findings suggest that children actively interpret and negotiate illness experiences in ways shaped by developmental priorities, lived experiences, and interactions with families and healthcare professionals. Integrating developmentally appropriate meaning‐making support into serious illness communication and pediatric palliative care may strengthen children's coping, participation, and psychosocial well‐being across the illness trajectory.

## Author Contributions

Yixuan Wang conceptualized and designed the study, developed the search strategy, conducted the literature search, screening, and data extraction, led the data analysis, drafted the original manuscript, and critically reviewed and revised the manuscript. Cheng Jin conducted the literature screening and data extraction, contributed to the data analysis, participated in manuscript development, and critically reviewed and revised the manuscript. Jennifer Currin‐McCulloch and David MacPhee supervised and contributed to the data analysis, provided conceptual guidance, participated in manuscript development, and critically reviewed and revised the manuscript. Abby R. Rosenberg provided conceptual guidance, participated in manuscript development, and critically reviewed and revised the manuscript for important intellectual content. Yijia Yang, Zhenrong Su, Peichen Liu, Jiameng Wang, and Sydney Pryor conducted the literature screening and data extraction and reviewed the manuscript and provided feedback. All authors approved the final manuscript and agree to be accountable for all aspects of the work.

## Funding

The authors have nothing to report.

## Ethics Statement

The authors have nothing to report.

## Conflicts of Interest

The authors declare no conflicts of interest.

## Supporting information


Supporting Information S1

## Data Availability

The authors have nothing to report.
