# Supplementary material for: Not Too Young to Understand: Meaning Making From the Perspectives of School‐Aged Children With Cancer—A Scoping Review
Source: Psychooncology. 2026 Sep 2;35(9):e70592. doi: 10.1002/pon.70592 (PMC13537027; doi:10.1002/pon.70592)
Supplement: Supplementary file 1 [file PON-35-e70592-s001.docx]

**Research question: What is known about the meaning making processes of children and adolescents with cancer through their perspectives?**

**Table S1**

*Summary of Search Results by Database*

| Database | Search Result (Total) | Search Date |
| --- | --- | --- |
| APA PsycInfo (EBSCOhost) | 776 | 3/17/2025 |
| CINAHL (EBSCOhost) | 3,333 | 3/18/2025 |
| MEDLINE (EBSCOhost) | 3,513 | 3/18/2025 |
| Web of Science | 1,016 | 3/18/2025 |
| Total results (duplicates included) | 8,638 | 3/18/2025 |
| Total results (duplicates removed) | 6,058 | 3/18/2025 |

**Table S2**

*Core Concepts and Search Terms*

| Core Concept | Searching Term |
| --- | --- |
| Children and adolescents | "newborn*" OR "neonate*" OR "infan*" OR "baby*" OR "babies" OR "toddler*" OR "preschool*" OR "nursery school*" OR "kindergarten*" OR "school-age*" OR "early age*" OR "elementary school*" OR "primary school*" OR "tween*" OR "preteen*" OR "prepuberty" OR "prepubescent*" OR "prepubertal*" OR "child*" OR "middle school*" OR "teen*" OR "puberty" OR "pubescent*" OR "pubertal*" OR "adolescen*" OR "high school*" OR "youth*" OR "pediatric" OR "paediatric" |
| Cancer | "cancer" OR "cancer survivor*" OR "neoplasm*" OR "oncolog*" OR "malignant tumor*" OR "malignant tumour*" OR "leukemia" OR "leukaemia" OR "brain tumor*" OR "brain tumour*"OR "neuroblastoma" OR "wilms tumor*" OR "wilms tumour*" OR "retinoblastoma" OR "yolk sac tumor*" OR "yolk sac tumour*" OR "rhabdomyosarcoma" OR "osteosarcoma" OR "sarcoma*" OR "lymphoma" |
| Meaning-making process | "meaning making" OR "sense making" OR "meaning construction" OR "meaning reconstruction" OR "global meaning" OR "global belief*" OR "situational meaning" OR "positive psycholog*" OR "positive meaning*" OR "positive appraisal*" OR "positive interpretation" OR "positive reappraisal*" OR "positive reinterpretation" OR "appraised meaning" OR "perceived meaning" OR (meaning N3 made) OR "meaning-centered" OR "meaning-centred" OR "meaning-based" OR "meaning-focused" OR "meaning-oriented" OR "meaning-related" OR "meaningful*" OR "meaningless*" OR (meaning N3 violat*) OR "meaning in life" OR (meaning* N3 disease*) OR (meaning* N3 illness*) OR (meaning* N3 cancer*) OR (meaning* N3 survivor*) OR (meaning* N3 patient*) OR (religious N3 meaning*) OR (spiritual N3 meaning*) OR (social N3 meaning*) OR (cultural N3 meaning*) OR (sociocultural N3 meaning*) OR (existential N3 meaning*) OR "meaning*" OR "significance" OR "attitude*" OR "belief*" OR "value" OR "worldview" OR "world view" OR "faith*" OR "knowledge" OR "religio*" OR "spiritual*" OR "existen*" OR "identit*" OR "self-concept" OR "fair*" OR "just*" OR "motivat*" OR "goal*" OR "desir*" OR "preference*" OR "sense of meaning*" OR "sense of purpose" OR "sense of self*" OR "sense of agency" OR "sense of connectedness" OR "sense of belonging" OR "lived experience*" OR "stress*" OR "trauma*" OR "advers*" OR "attribut*" OR "appraise*" OR "appraisal*" OR "control*" OR "self-congruence" OR "sense of coherence" OR "self-efficacy" OR "cognitive dissonance" OR "disequilibrium" OR "discrepan*" OR "dissatisf*" OR "distress*" OR "doubt*" OR "fear*" OR "grief" OR "hope*" OR "suffering" OR "uncertain*" OR "anxiety" OR "anxious*" OR "depress*" OR "intrusive thought*" OR "accommodation" OR "assimilation" OR "aware*" OR "concept*" OR "expect*" OR "strateg*" OR "perceive*" OR "perception" OR "reflect*" OR "schema*" OR "coping" OR "understand*" OR "interpret*" OR "comprehen*" OR "emotional* process*" OR "cognitive* process*" OR "adjustment" OR "adaptation" OR "adaptive" OR "acceptance" OR "regulation" OR "resilienc*" OR "post-traumatic growth" OR "cancer-related growth" OR "cognitive development" OR "emotional development" OR "psychological development" OR "social development" OR "psychosocial development" OR "identity development" OR "refram*" OR "reappraisal" OR "restor*" OR "reinterpret*" OR "compassio*" OR "empath*" OR "gratitude" OR "grateful" OR "optimis*" OR "satisf*" OR "happiness" OR "peace*" OR "emotional well-being" OR "psychological well-being" OR "mental well-being" OR "social well-being" OR "psychosocial well-being" OR "spiritual well-being" OR "existential well-being" OR "subjective well-being" |
| Child and adolescent perspective | "child-led" OR "adolescent-led" OR "youth-led" OR "child-centered" OR "adolescent-centered" OR "youth-centered" OR "child-centred" OR "adolescent-centred" OR "youth-centred" OR "child-focused" OR "adolescent-focused" OR "youth-focused" OR (child* N3 voice*) OR (adolescent* N3 voice*) OR (youth* N3 voice*) OR (child* N3 perspective*) OR (adolescent* N3 perspective*) OR (youth* N3 perspective*) OR "self-report*" OR "patient-report*" OR "self-assess*" OR "self-evaluat*" OR "interview*" OR "focus group" OR "diar*" OR "journal*" OR "drawing*" OR "draw-and-tell" OR "draw-and-write" OR "painting*" OR "music*" OR "photo*" OR "storytelling" OR "life stor*" OR "play therapy" OR "expressive writing" OR "observ*" OR "narrative*" OR "participatory" OR "advisory board" OR "autobiography" |

**Table S3**

*Subject Headings for the APA PsycInfo Database (EBSCOhost)*

| Concept | Subject Headings |
| --- | --- |
| Children and adolescents | neonatal (birth-1 mo) OR infancy (2-23 mo) OR preschool age (2-5 yrs) OR school age (6-12 yrs) OR childhood (birth-12 yrs) OR adolescence (13-17 yrs) |
| Cancer | Neoplasms OR Breast Neoplasms OR Childhood Neoplasms OR Digestive System Neoplasms OR Colorectal Neoplasms OR Endocrine Neoplasms OR Leukemias OR Lung Neoplasms OR Metastasis OR Nervous System Neoplasms OR Brain Neoplasms OR Glioma OR Skin Neoplasms OR Melanoma OR Terminal Cancer OR Oncology |
| Meaning making process | Meaning OR Meaningfulness OR Attitudes OR Adolescent Attitudes OR Child Attitudes OR Death Attitudes OR Health Attitudes OR Explicit Attitudes OR Implicit Attitudes OR Mental Health (Attitudes Toward) OR Mental Illness (Attitudes Toward) OR Physical Illness (Attitudes Toward) OR Values OR Dignity OR Personal Values OR World View OR Religious Beliefs OR Faith OR Spirituality OR Health Belief Model OR Health Knowledge OR Health Locus of Control OR Positive Psychology OR Identity Crisis OR Identity Formation OR Motivation OR Goals OR Goal Orientation OR Goal Setting OR Subjectivity OR Sense of Purpose OR Experiences (Events) OR Life Experiences OR Life Changes OR Early Experience OR Lived Experience OR Stress OR Acute Stress OR Chronic Stress OR Perceived Stress OR Physiological Stress OR Posttraumatic Stress OR Psychological Stress OR Social Stress OR Trauma OR Developmental Trauma OR Emotional Trauma OR Traumatic Experiences OR Adversity OR Childhood Adversity OR Attribution OR Cognitive Appraisal OR Perceived Control OR Self-Affirmation OR Self-Congruence OR Sense of Coherence OR Self-Efficacy OR Cognitive Dissonance OR Emotional States OR Dissatisfaction OR Distress OR Doubt OR Fear OR Grief OR Hopelessness OR Negative Emotions OR Suffering OR Uncertainty OR Cognitive Process OR Abstraction OR Accommodation (Cognitive Process) OR Assimilation (Cognitive Process) OR Awareness OR Body Awareness OR Health Awareness OR Situation Awareness OR Cognition OR Cognitions OR Concepts OR Expectations OR Cognitive Control OR Cognitive Strategies OR Metacognition OR Perception OR Self-Perception OR Self-Acceptance OR Self-Compassion OR Self-Confidence OR Self-Deception OR Self-Esteem OR Self-Forgiveness OR Self-Knowledge OR Self-Reference OR Self-Regard OR Self-Reflection OR Self-Worth OR Problem Solving OR Psychological Detachment OR Psychological Engagement OR Reasoning OR Rumination (Cognitive Process) OR Schema OR Cognitive Strategies OR Thinking OR Comprehension OR Coping Behavior OR Coping Style OR Self-Control OR Emotional Process OR Emotional Control OR Emotional Regulation OR Emotional Adjustment OR Adaptive Behavior OR Resilience (Psychological) OR Posttraumatic Growth OR Psychological Development OR Psychosocial Development OR Childhood Development OR Neonatal Development OR Infant Development OR Early Childhood Development OR Adolescent Development OR Self-Regulation OR Emotional Well Being OR Social Well Being OR Spiritual Well Being OR Subjective Well Being OR Compassion OR Desire OR Empathy OR Gratitude OR Hope OR Optimism OR Positive Emotions OR Satisfaction OR Happiness |
| Child and adolescent perspective | Self-Report OR Self-Evaluation OR Interviews OR Focus Group Interview OR Semi-Structured Interview OR Storytelling OR Digital Storytelling OR Focus Group OR Journal Writing OR Art Therapy OR Drawing OR Painting (Art) OR Photographic Art OR Photographs OR Play Therapy OR Music Therapy OR Self-Expression OR Creative Writing OR Observation Methods OR Direct Observation OR Participant Observation OR Narratives OR Action Research OR Autobiography OR Community-Based Participatory Research |

**Table S4**

*Search Strategy and Results for the APA PsycInfo Database (EBSCOhost)*

| No. | Query | Search Result |
| --- | --- | --- |
| S1 | AG (neonatal (birth-1 mo) OR infancy (2-23 mo) OR preschool age (2-5 yrs) OR school age (6-12 yrs) OR childhood (birth-12 yrs) OR adolescence (13-17 yrs)) | 937,276 |
| S2 | TI ( "newborn*" OR "neonate*" OR "infan*" OR "baby*" OR "babies" OR "toddler*" OR "preschool*" OR "nursery school*" OR "kindergarten*" OR "school-age*" OR "early age*" OR "elementary school*" OR "primary school*" OR "tween*" OR "preteen*" OR "prepuberty" OR "prepubescent*" OR "prepubertal*" OR "child*" OR "middle school*" OR "teen*" OR "puberty" OR "pubescent*" OR "pubertal*" OR "adolescen*" OR "high school*" OR "youth*" OR "pediatric" OR "paediatric" ) OR AB ( "newborn*" OR "neonate*" OR "infan*" OR "baby*" OR "babies" OR "toddler*" OR "preschool*" OR "nursery school*" OR "kindergarten*" OR "school-age*" OR "early age*" OR "elementary school*" OR "primary school*" OR "tween*" OR "preteen*" OR "prepuberty" OR "prepubescent*" OR "prepubertal*" OR "child*" OR "middle school*" OR "teen*" OR "puberty" OR "pubescent*" OR "pubertal*" OR "adolescen*" OR "high school*" OR "youth*" OR "pediatric" OR "paediatric" ) | 1,223,439 |
| S3 | S1 OR S2 | 1,441,211 |
| S4 | DE (Neoplasms OR Breast Neoplasms OR Childhood Neoplasms OR Digestive System Neoplasms OR Colorectal Neoplasms OR Endocrine Neoplasms OR Leukemias OR Lung Neoplasms OR Metastasis OR Nervous System Neoplasms OR Brain Neoplasms OR Glioma OR Skin Neoplasms OR Melanoma OR Terminal Cancer OR Oncology) | 74,071 |
| S5 | TI ( "cancer" OR "cancer survivor*" OR "neoplasm*" OR "oncolog*" OR "malignant tumor*" OR "malignant tumour*" OR "leukemia" OR "leukaemia" OR "brain tumor*" OR "brain tumour*"OR "neuroblastoma" OR "wilms tumor*" OR "wilms tumour*" OR "retinoblastoma" OR "yolk sac tumor*" OR "yolk sac tumour*" OR "rhabdomyosarcoma" OR "osteosarcoma" OR "sarcoma*" OR "lymphoma" ) OR AB ( "cancer" OR "cancer survivor*" OR "neoplasm*" OR "oncolog*" OR "malignant tumor*" OR "malignant tumour*" OR "leukemia" OR "leukaemia" OR "brain tumor*" OR "brain tumour*"OR "neuroblastoma" OR "wilms tumor*" OR "wilms tumour*" OR "retinoblastoma" OR "yolk sac tumor*" OR "yolk sac tumour*" OR "rhabdomyosarcoma" OR "osteosarcoma" OR "sarcoma*" OR "lymphoma" ) | 87,071 |
| S6 | S4 OR S5 | 96,300 |
| S7 | AG ( neonatal (birth-1 mo) OR infancy (2-23 mo) OR preschool age (2-5 yrs) OR school age (6-12 yrs) OR childhood (birth-12 yrs) OR adolescence (13-17 yrs) ) AND ( DE (Meaning OR Meaningfulness OR Attitudes OR Adolescent Attitudes OR Child Attitudes OR Death Attitudes OR Health Attitudes OR Explicit Attitudes OR Implicit Attitudes OR Mental Health (Attitudes Toward) OR Mental Illness (Attitudes Toward) OR Physical Illness (Attitudes Toward) OR Values OR Dignity OR Personal Values OR World View OR Religious Beliefs OR Faith OR Spirituality OR Health Belief Model OR Health Knowledge OR Health Locus of Control OR Positive Psychology OR Identity Crisis OR Identity Formation OR Motivation OR Goals OR Goal Orientation OR Goal Setting OR Subjectivity OR Sense of Purpose OR Experiences (Events) OR Life Experiences OR Life Changes OR Early Experience OR Lived Experience OR Stress OR Acute Stress OR Chronic Stress OR Perceived Stress OR Physiological Stress OR Posttraumatic Stress OR Psychological Stress OR Social Stress OR Trauma OR Developmental Trauma OR Emotional Trauma OR Traumatic Experiences OR Adversity OR Childhood Adversity OR Attribution OR Cognitive Appraisal OR Perceived Control OR Self-Affirmation OR Self-Congruence OR Sense of Coherence OR Self-Efficacy OR Cognitive Dissonance OR Emotional States OR Dissatisfaction OR Distress OR Doubt OR Fear OR Grief OR Hopelessness OR Negative Emotions OR Suffering OR Uncertainty OR Cognitive Process OR Abstraction OR Accommodation (Cognitive Process) OR Assimilation (Cognitive Process) OR Awareness OR Body Awareness OR Health Awareness OR Situation Awareness OR Cognition OR Cognitions OR Concepts OR Expectations OR Cognitive Control OR Cognitive Strategies OR Metacognition OR Perception OR Self-Perception OR Self-Acceptance OR Self-Compassion OR Self-Confidence OR Self-Deception OR Self-Esteem OR Self-Forgiveness OR Self-Knowledge OR Self-Reference OR Self-Regard OR Self-Reflection OR Self-Worth OR Problem Solving OR Psychological Detachment OR Psychological Engagement OR Reasoning OR Rumination (Cognitive Process) OR Schema OR Cognitive Strategies OR Thinking OR Comprehension OR Coping Behavior OR Coping Style OR Self-Control OR Emotional Process OR Emotional Control OR Emotional Regulation OR Emotional Adjustment OR Adaptive Behavior OR Resilience (Psychological) OR Posttraumatic Growth OR Psychological Development OR Psychosocial Development OR Childhood Development OR Neonatal Development OR Infant Development OR Early Childhood Development OR Adolescent Development OR Self-Regulation OR Emotional Well Being OR Social Well Being OR Spiritual Well Being OR Subjective Well Being OR Compassion OR Desire OR Empathy OR Gratitude OR Hope OR Optimism OR Positive Emotions OR Satisfaction OR Happiness) ) | 334,797 |
| S8 | TI ( ("meaning making" OR "sense making" OR "meaning construction" OR "meaning reconstruction" OR "global meaning" OR "global belief*" OR "situational meaning" OR "positive psycholog*" OR "positive meaning*" OR "positive appraisal*" OR "positive interpretation" OR "positive reappraisal*" OR "positive reinterpretation" OR "appraised meaning" OR "perceived meaning" OR (meaning N3 made) OR "meaning-centered" OR "meaning-centred" OR "meaning-based" OR "meaning-focused" OR "meaning-oriented" OR "meaning-related" OR "meaningful*" OR "meaningless*" OR (meaning N3 violat*) OR "meaning in life" OR (meaning* N3 disease*) OR (meaning* N3 illness*) OR (meaning* N3 cancer*) OR (meaning* N3 survivor*) OR (meaning* N3 patient*) OR (religious N3 meaning*) OR (spiritual N3 meaning*) OR (social N3 meaning*) OR (cultural N3 meaning*) OR (sociocultural N3 meaning*) OR (existential N3 meaning*)) OR (("newborn*" OR "neonate*" OR "infan*" OR "baby*" OR "babies" OR "toddler*" OR "preschool*" OR "nursery school*" OR "kindergarten*" OR "school-age*" OR "early age*" OR "elementary school*" OR "primary school*" OR "tween*" OR "preteen*" OR "prepuberty" OR "prepubescent*" OR "prepubertal*" OR "child*" OR "middle school*" OR "teen*" OR "puberty" OR "pubescent*" OR "pubertal*" OR "adolescen*" OR "high school*" OR "youth*" OR "pediatric" OR "paediatric") N3 ("meaning*" OR "significance" OR "attitude*" OR "belief*" OR "value" OR "worldview" OR "world view" OR "faith*" OR "knowledge" OR "religio*" OR "spiritual*" OR "existen*" OR "identit*" OR "self-concept" OR "fair*" OR "just*" OR "motivat*" OR "goal*" OR "desir*" OR "preference*" OR "sense of meaning*" OR "sense of purpose" OR "sense of self*" OR "sense of agency" OR "sense of connectedness" OR "sense of belonging" OR "lived experience*" OR "stress*" OR "trauma*" OR "advers*" OR "attribut*" OR "appraise*" OR "appraisal*" OR "control*" OR "self-congruence" OR "sense of coherence" OR "self-efficacy" OR "cognitive dissonance" OR "disequilibrium" OR "discrepan*" OR "dissatisf*" OR "distress*" OR "doubt*" OR "fear*" OR "grief" OR "hope*" OR "suffering" OR "uncertain*" OR "anxiety" OR "anxious*" OR "depress*" OR "intrusive thought*" OR "accommodation" OR "assimilation" OR "aware*" OR "concept*" OR "expect*" OR "strateg*" OR "perceive*" OR "perception" OR "reflect*" OR "schema*" OR "coping" OR "understand*" OR "interpret*" OR "comprehen*" OR "emotional* process*" OR "cognitive* process*" OR "adjustment" OR "adaptation" OR "adaptive" OR "acceptance" OR "regulation" OR "resilienc*" OR "post-traumatic growth" OR "cancer-related growth" OR "cognitive development" OR "emotional development" OR "psychological development" OR "social development" OR "psychosocial development" OR "identity development" OR "refram*" OR "reappraisal" OR "restor*" OR "reinterpret*" OR "compassio*" OR "empath*" OR "gratitude" OR "grateful" OR "optimis*" OR "satisf*" OR "happiness" OR "peace*" OR "emotional well-being" OR "psychological well-being" OR "mental well-being" OR "social well-being" OR "psychosocial well-being" OR "spiritual well-being" OR "existential well-being" OR "subjective well-being")) ) OR AB ( ("meaning making" OR "sense making" OR "meaning construction" OR "meaning reconstruction" OR "global meaning" OR "global belief*" OR "situational meaning" OR "positive psycholog*" OR "positive meaning*" OR "positive appraisal*" OR "positive interpretation" OR "positive reappraisal*" OR "positive reinterpretation" OR "appraised meaning" OR "perceived meaning" OR (meaning N3 made) OR "meaning-centered" OR "meaning-centred" OR "meaning-based" OR "meaning-focused" OR "meaning-oriented" OR "meaning-related" OR "meaningful*" OR "meaningless*" OR (meaning N3 violat*) OR "meaning in life" OR (meaning* N3 disease*) OR (meaning* N3 illness*) OR (meaning* N3 cancer*) OR (meaning* N3 survivor*) OR (meaning* N3 patient*) OR (religious N3 meaning*) OR (spiritual N3 meaning*) OR (social N3 meaning*) OR (cultural N3 meaning*) OR (sociocultural N3 meaning*) OR (existential N3 meaning*)) OR (("newborn*" OR "neonate*" OR "infan*" OR "baby*" OR "babies" OR "toddler*" OR "preschool*" OR "nursery school*" OR "kindergarten*" OR "school-age*" OR "early age*" OR "elementary school*" OR "primary school*" OR "tween*" OR "preteen*" OR "prepuberty" OR "prepubescent*" OR "prepubertal*" OR "child*" OR "middle school*" OR "teen*" OR "puberty" OR "pubescent*" OR "pubertal*" OR "adolescen*" OR "high school*" OR "youth*" OR "pediatric" OR "paediatric") N3 ("meaning*" OR "significance" OR "attitude*" OR "belief*" OR "value" OR "worldview" OR "world view" OR "faith*" OR "knowledge" OR "religio*" OR "spiritual*" OR "existen*" OR "identit*" OR "self-concept" OR "fair*" OR "just*" OR "motivat*" OR "goal*" OR "desir*" OR "preference*" OR "sense of meaning*" OR "sense of purpose" OR "sense of self*" OR "sense of agency" OR "sense of connectedness" OR "sense of belonging" OR "lived experience*" OR "stress*" OR "trauma*" OR "advers*" OR "attribut*" OR "appraise*" OR "appraisal*" OR "control*" OR "self-congruence" OR "sense of coherence" OR "self-efficacy" OR "cognitive dissonance" OR "disequilibrium" OR "discrepan*" OR "dissatisf*" OR "distress*" OR "doubt*" OR "fear*" OR "grief" OR "hope*" OR "suffering" OR "uncertain*" OR "anxiety" OR "anxious*" OR "depress*" OR "intrusive thought*" OR "accommodation" OR "assimilation" OR "aware*" OR "concept*" OR "expect*" OR "strateg*" OR "perceive*" OR "perception" OR "reflect*" OR "schema*" OR "coping" OR "understand*" OR "interpret*" OR "comprehen*" OR "emotional* process*" OR "cognitive* process*" OR "adjustment" OR "adaptation" OR "adaptive" OR "acceptance" OR "regulation" OR "resilienc*" OR "post-traumatic growth" OR "cancer-related growth" OR "cognitive development" OR "emotional development" OR "psychological development" OR "social development" OR "psychosocial development" OR "identity development" OR "refram*" OR "reappraisal" OR "restor*" OR "reinterpret*" OR "compassio*" OR "empath*" OR "gratitude" OR "grateful" OR "optimis*" OR "satisf*" OR "happiness" OR "peace*" OR "emotional well-being" OR "psychological well-being" OR "mental well-being" OR "social well-being" OR "psychosocial well-being" OR "spiritual well-being" OR "existential well-being" OR "subjective well-being")) ) | 440,829 |
| S9 | S7 OR S8 | 645,473 |
| S10 | AG ( "newborn*" OR "neonate*" OR "infan*" OR "baby*" OR "babies" OR "toddler*" OR "preschool*" OR "nursery school*" OR "kindergarten*" OR "school-age*" OR "early age*" OR "elementary school*" OR "primary school*" OR "tween*" OR "preteen*" OR "prepuberty" OR "prepubescent*" OR "prepubertal*" OR "child*" OR "middle school*" OR "teen*" OR "puberty" OR "pubescent*" OR "pubertal*" OR "adolescen*" OR "high school*" OR "youth*" OR "pediatric" OR "paediatric" ) AND ( DE (Self-Report OR Self-Evaluation OR Interviews OR Focus Group Interview OR Semi-Structured Interview OR Storytelling OR Digital Storytelling OR Focus Group OR Journal Writing OR Art Therapy OR Drawing OR Painting (Art) OR Photographic Art OR Photographs OR Play Therapy OR Music Therapy OR Self-Expression OR Creative Writing OR Observation Methods OR Direct Observation OR Participant Observation OR Narratives OR Action Research OR Autobiography OR Community-Based Participatory Research) ) | 28,577 |
| S11 | TI ( ("child-led" OR "adolescent-led" OR "youth-led" OR "child-centered" OR "adolescent-centered" OR "youth-centered" OR "child-centred" OR "adolescent-centred" OR "youth-centred" OR "child-focused" OR "adolescent-focused" OR "youth-focused" OR (child* N3 voice*) OR (adolescent* N3 voice*) OR (youth* N3 voice*) OR (child* N3 perspective*) OR (adolescent* N3 perspective*) OR (youth* N3 perspective*)) OR (("newborn*" OR "neonate*" OR "infan*" OR "baby*" OR "babies" OR "toddler*" OR "preschool*" OR "nursery school*" OR "kindergarten*" OR "school-age*" OR "early age*" OR "elementary school*" OR "primary school*" OR "tween*" OR "preteen*" OR "prepuberty" OR "prepubescent*" OR "prepubertal*" OR "child*" OR "middle school*" OR "teen*" OR "puberty" OR "pubescent*" OR "pubertal*" OR "adolescen*" OR "high school*" OR "youth*" OR "pediatric" OR "paediatric") N3 ("self-report*" OR "patient-report*" OR "self-assess*" OR "self-evaluat*" OR "interview*" OR "focus group" OR "diar*" OR "journal*" OR "drawing*" OR "draw-and-tell" OR "draw-and-write" OR "painting*" OR "music*" OR "photo*" OR "storytelling" OR "life stor*" OR "play therapy" OR "expressive writing" OR "observ*" OR "narrative*" OR "participatory" OR "advisory board" OR "autobiography")) ) OR AB ( ("child-led" OR "adolescent-led" OR "youth-led" OR "child-centered" OR "adolescent-centered" OR "youth-centered" OR "child-centred" OR "adolescent-centred" OR "youth-centred" OR "child-focused" OR "adolescent-focused" OR "youth-focused" OR (child* N3 voice*) OR (adolescent* N3 voice*) OR (youth* N3 voice*) OR (child* N3 perspective*) OR (adolescent* N3 perspective*) OR (youth* N3 perspective*)) OR (("newborn*" OR "neonate*" OR "infan*" OR "baby*" OR "babies" OR "toddler*" OR "preschool*" OR "nursery school*" OR "kindergarten*" OR "school-age*" OR "early age*" OR "elementary school*" OR "primary school*" OR "tween*" OR "preteen*" OR "prepuberty" OR "prepubescent*" OR "prepubertal*" OR "child*" OR "middle school*" OR "teen*" OR "puberty" OR "pubescent*" OR "pubertal*" OR "adolescen*" OR "high school*" OR "youth*" OR "pediatric" OR "paediatric") N3 ("self-report*" OR "patient-report*" OR "self-assess*" OR "self-evaluat*" OR "interview*" OR "focus group" OR "diar*" OR "journal*" OR "drawing*" OR "draw-and-tell" OR "draw-and-write" OR "painting*" OR "music*" OR "photo*" OR "storytelling" OR "life stor*" OR "play therapy" OR "expressive writing" OR "observ*" OR "narrative*" OR "participatory" OR "advisory board" OR "autobiography")) ) | 88,500 |
| S12 | S10 OR S11 | 107,371 |
| S13 | S3 AND S6 AND S9 AND S12 | 776 |

**Table S5**

*Subject Headings for the CINAHL Database (EBSCOhost)*

| Concept | Subject Headings |
| --- | --- |
| Children and adolescents | (MH "Infant+") OR (MH "Child+") OR (MH "Adolescence+") |
| Cancer | (MH "Neoplasms+") OR (MH "Oncology+") OR (MH "Oncologic Care+") OR (MH "Cancer Survivors") |
| Meaning-making processes | (MH "Life Purpose") OR (MH "Positive Psychology") OR (MH "Attitude to Death+") OR (MH "Attitude to Health+") OR (MH "Attitude to Illness+") OR (MH "Attitude to Life") OR (MH "Patient Attitudes") OR (MH "Personal Values") OR (MH "Religion and Religions+") OR (MH "Health Belief Model") OR (MH "Health Knowledge") OR (MH "Identity Crisis") OR (MH "Motivation") OR (MH "Goals and Objectives+") OR (MH "Self Concept+") OR (MH "Life Experiences") OR (MH "Life Change Events") OR (MH "Catastrophic Illness") OR (MH "Stress, Psychological") OR (MH "Subjective Stress") OR (MH "Trauma") OR (MH "Adverse Childhood Experiences") OR (MH "Control (Psychology)+") OR (MH "Mental Processes") OR (MH "Cognition") OR (MH "Cognitive Dissonance") OR (MH "Rumination (Cognition)") OR (MH "Intention") OR (MH "Personal Growth") OR (MH "Problem Solving") OR (MH "Perception+") OR (MH "Reflection") OR (MH "Theory of Mind") OR (MH "Thinking+") OR (MH "Emotions") OR (MH "Compassion") OR (MH "Emotional Regulation") OR (MH "Fear") OR (MH "Hope") OR (MH "Hopelessness") OR (MH "Psychological Distress") OR (MH "Suffering") OR (MH "Empathy") OR (MH "Optimism") OR (MH "Hardiness") OR (MH "Uncertainty") OR (MH "Coping+") OR (MH "Adaptation, Psychological+") OR (MH "Adolescent Development") OR (MH "Child Development") OR (MH "Emotional Maturity") OR (MH "Infant Development") OR (MH "Psychological Well-Being") OR (MH "Happiness") |
| Child and adolescent perspective | (MH "Self Report+") OR (MH "Self Assessment") OR (MH "Interviews+") OR (MH "Focus Groups") OR (MH "Diaries") OR (MH "Drawing") OR (MH "Autobiographies") OR (MH "Storytelling") OR (MH "Narratives+") OR (MH "Writing") OR (MH "Observational Methods") OR (MH "Action Research") OR (MH "Art Therapy") OR (MH "Play Therapy") OR (MH "Music Therapy") |

**Table S6**

*Search Strategy and Results for the CINAHL Database (EBSCOhost)*

| No. | Query | Search Result |
| --- | --- | --- |
| S1 | (MH "Infant+") OR (MH "Child+") OR (MH "Adolescence+") | 1,151,366 |
| S2 | TI ( "newborn*" OR "neonate*" OR "infan*" OR "baby*" OR "babies" OR "toddler*" OR "preschool*" OR "nursery school*" OR "kindergarten*" OR "school-age*" OR "early age*" OR "elementary school*" OR "primary school*" OR "tween*" OR "preteen*" OR "prepuberty" OR "prepubescent*" OR "prepubertal*" OR "child*" OR "middle school*" OR "teen*" OR "puberty" OR "pubescent*" OR "pubertal*" OR "adolescen*" OR "high school*" OR "youth*" OR "pediatric" OR "paediatric" ) OR AB ( "newborn*" OR "neonate*" OR "infan*" OR "baby*" OR "babies" OR "toddler*" OR "preschool*" OR "nursery school*" OR "kindergarten*" OR "school-age*" OR "early age*" OR "elementary school*" OR "primary school*" OR "tween*" OR "preteen*" OR "prepuberty" OR "prepubescent*" OR "prepubertal*" OR "child*" OR "middle school*" OR "teen*" OR "puberty" OR "pubescent*" OR "pubertal*" OR "adolescen*" OR "high school*" OR "youth*" OR "pediatric" OR "paediatric" ) | 998,928 |
| S3 | S1 OR S2 | 1,483,287 |
| S4 | (MH "Neoplasms+") OR (MH "Oncology+") OR (MH "Oncologic Care+") OR (MH "Cancer Survivors") | 687,381 |
| S5 | TI ( "cancer" OR "cancer survivor*" OR "neoplasm*" OR "oncolog*" OR "malignant tumor*" OR "malignant tumour*" OR "leukemia" OR "leukaemia" OR "brain tumor*" OR "brain tumour*"OR "neuroblastoma" OR "wilms tumor*" OR "wilms tumour*" OR "retinoblastoma" OR "yolk sac tumor*" OR "yolk sac tumour*" OR "rhabdomyosarcoma" OR "osteosarcoma" OR "sarcoma*" OR "lymphoma" ) OR AB ( "cancer" OR "cancer survivor*" OR "neoplasm*" OR "oncolog*" OR "malignant tumor*" OR "malignant tumour*" OR "leukemia" OR "leukaemia" OR "brain tumor*" OR "brain tumour*"OR "neuroblastoma" OR "wilms tumor*" OR "wilms tumour*" OR "retinoblastoma" OR "yolk sac tumor*" OR "yolk sac tumour*" OR "rhabdomyosarcoma" OR "osteosarcoma" OR "sarcoma*" OR "lymphoma" ) | 624,317 |
| S6 | S4 OR S5 | 889,170 |
| S7 | ( (MH "Infant+") OR (MH "Child+") OR (MH "Adolescence+") ) AND ( (MH "Life Purpose") OR (MH "Positive Psychology") OR (MH "Attitude to Death+") OR (MH "Attitude to Health+") OR (MH "Attitude to Illness+") OR (MH "Attitude to Life") OR (MH "Patient Attitudes") OR (MH "Personal Values") OR (MH "Religion and Religions+") OR (MH "Health Belief Model") OR (MH "Health Knowledge") OR (MH "Identity Crisis") OR (MH "Motivation") OR (MH "Goals and Objectives+") OR (MH "Self Concept+") OR (MH "Life Experiences") OR (MH "Life Change Events") OR (MH "Catastrophic Illness") OR (MH "Stress, Psychological") OR (MH "Subjective Stress") OR (MH "Trauma") OR (MH "Adverse Childhood Experiences") OR (MH "Control (Psychology)+") OR (MH "Mental Processes") OR (MH "Cognition") OR (MH "Cognitive Dissonance") OR (MH "Rumination (Cognition)") OR (MH "Intention") OR (MH "Personal Growth") OR (MH "Problem Solving") OR (MH "Perception+") OR (MH "Reflection") OR (MH "Theory of Mind") OR (MH "Thinking+") OR (MH "Emotions") OR (MH "Compassion") OR (MH "Emotional Regulation") OR (MH "Fear") OR (MH "Hope") OR (MH "Hopelessness") OR (MH "Psychological Distress") OR (MH "Suffering") OR (MH "Empathy") OR (MH "Optimism") OR (MH "Hardiness") OR (MH "Uncertainty") OR (MH "Coping+") OR (MH "Adaptation, Psychological+") OR (MH "Adolescent Development") OR (MH "Child Development") OR (MH "Emotional Maturity") OR (MH "Infant Development") OR (MH "Psychological Well-Being") OR (MH "Happiness") ) | 245,626 |
| S8 | TI ( ("meaning making" OR "sense making" OR "meaning construction" OR "meaning reconstruction" OR "global meaning" OR "global belief*" OR "situational meaning" OR "positive psycholog*" OR "positive meaning*" OR "positive appraisal*" OR "positive interpretation" OR "positive reappraisal*" OR "positive reinterpretation" OR "appraised meaning" OR "perceived meaning" OR (meaning N3 made) OR "meaning-centered" OR "meaning-centred" OR "meaning-based" OR "meaning-focused" OR "meaning-oriented" OR "meaning-related" OR "meaningful*" OR "meaningless*" OR (meaning N3 violat*) OR "meaning in life" OR (meaning* N3 disease*) OR (meaning* N3 illness*) OR (meaning* N3 cancer*) OR (meaning* N3 survivor*) OR (meaning* N3 patient*) OR (religious N3 meaning*) OR (spiritual N3 meaning*) OR (social N3 meaning*) OR (cultural N3 meaning*) OR (sociocultural N3 meaning*) OR (existential N3 meaning*)) OR (("newborn*" OR "neonate*" OR "infan*" OR "baby*" OR "babies" OR "toddler*" OR "preschool*" OR "nursery school*" OR "kindergarten*" OR "school-age*" OR "early age*" OR "elementary school*" OR "primary school*" OR "tween*" OR "preteen*" OR "prepuberty" OR "prepubescent*" OR "prepubertal*" OR "child*" OR "middle school*" OR "teen*" OR "puberty" OR "pubescent*" OR "pubertal*" OR "adolescen*" OR "high school*" OR "youth*" OR "pediatric" OR "paediatric") N3 ("meaning*" OR "significance" OR "attitude*" OR "belief*" OR "value" OR "worldview" OR "world view" OR "faith*" OR "knowledge" OR "religio*" OR "spiritual*" OR "existen*" OR "identit*" OR "self-concept" OR "fair*" OR "just*" OR "motivat*" OR "goal*" OR "desir*" OR "preference*" OR "sense of meaning*" OR "sense of purpose" OR "sense of self*" OR "sense of agency" OR "sense of connectedness" OR "sense of belonging" OR "lived experience*" OR "stress*" OR "trauma*" OR "advers*" OR "attribut*" OR "appraise*" OR "appraisal*" OR "control*" OR "self-congruence" OR "sense of coherence" OR "self-efficacy" OR "cognitive dissonance" OR "disequilibrium" OR "discrepan*" OR "dissatisf*" OR "distress*" OR "doubt*" OR "fear*" OR "grief" OR "hope*" OR "suffering" OR "uncertain*" OR "anxiety" OR "anxious*" OR "depress*" OR "intrusive thought*" OR "accommodation" OR "assimilation" OR "aware*" OR "concept*" OR "expect*" OR "strateg*" OR "perceive*" OR "perception" OR "reflect*" OR "schema*" OR "coping" OR "understand*" OR "interpret*" OR "comprehen*" OR "emotional* process*" OR "cognitive* process*" OR "adjustment" OR "adaptation" OR "adaptive" OR "acceptance" OR "regulation" OR "resilienc*" OR "post-traumatic growth" OR "cancer-related growth" OR "cognitive development" OR "emotional development" OR "psychological development" OR "social development" OR "psychosocial development" OR "identity development" OR "refram*" OR "reappraisal" OR "restor*" OR "reinterpret*" OR "compassio*" OR "empath*" OR "gratitude" OR "grateful" OR "optimis*" OR "satisf*" OR "happiness" OR "peace*" OR "emotional well-being" OR "psychological well-being" OR "mental well-being" OR "social well-being" OR "psychosocial well-being" OR "spiritual well-being" OR "existential well-being" OR "subjective well-being")) ) OR AB ( ("meaning making" OR "sense making" OR "meaning construction" OR "meaning reconstruction" OR "global meaning" OR "global belief*" OR "situational meaning" OR "positive psycholog*" OR "positive meaning*" OR "positive appraisal*" OR "positive interpretation" OR "positive reappraisal*" OR "positive reinterpretation" OR "appraised meaning" OR "perceived meaning" OR (meaning N3 made) OR "meaning-centered" OR "meaning-centred" OR "meaning-based" OR "meaning-focused" OR "meaning-oriented" OR "meaning-related" OR "meaningful*" OR "meaningless*" OR (meaning N3 violat*) OR "meaning in life" OR (meaning* N3 disease*) OR (meaning* N3 illness*) OR (meaning* N3 cancer*) OR (meaning* N3 survivor*) OR (meaning* N3 patient*) OR (religious N3 meaning*) OR (spiritual N3 meaning*) OR (social N3 meaning*) OR (cultural N3 meaning*) OR (sociocultural N3 meaning*) OR (existential N3 meaning*)) OR (("newborn*" OR "neonate*" OR "infan*" OR "baby*" OR "babies" OR "toddler*" OR "preschool*" OR "nursery school*" OR "kindergarten*" OR "school-age*" OR "early age*" OR "elementary school*" OR "primary school*" OR "tween*" OR "preteen*" OR "prepuberty" OR "prepubescent*" OR "prepubertal*" OR "child*" OR "middle school*" OR "teen*" OR "puberty" OR "pubescent*" OR "pubertal*" OR "adolescen*" OR "high school*" OR "youth*" OR "pediatric" OR "paediatric") N3 ("meaning*" OR "significance" OR "attitude*" OR "belief*" OR "value" OR "worldview" OR "world view" OR "faith*" OR "knowledge" OR "religio*" OR "spiritual*" OR "existen*" OR "identit*" OR "self-concept" OR "fair*" OR "just*" OR "motivat*" OR "goal*" OR "desir*" OR "preference*" OR "sense of meaning*" OR "sense of purpose" OR "sense of self*" OR "sense of agency" OR "sense of connectedness" OR "sense of belonging" OR "lived experience*" OR "stress*" OR "trauma*" OR "advers*" OR "attribut*" OR "appraise*" OR "appraisal*" OR "control*" OR "self-congruence" OR "sense of coherence" OR "self-efficacy" OR "cognitive dissonance" OR "disequilibrium" OR "discrepan*" OR "dissatisf*" OR "distress*" OR "doubt*" OR "fear*" OR "grief" OR "hope*" OR "suffering" OR "uncertain*" OR "anxiety" OR "anxious*" OR "depress*" OR "intrusive thought*" OR "accommodation" OR "assimilation" OR "aware*" OR "concept*" OR "expect*" OR "strateg*" OR "perceive*" OR "perception" OR "reflect*" OR "schema*" OR "coping" OR "understand*" OR "interpret*" OR "comprehen*" OR "emotional* process*" OR "cognitive* process*" OR "adjustment" OR "adaptation" OR "adaptive" OR "acceptance" OR "regulation" OR "resilienc*" OR "post-traumatic growth" OR "cancer-related growth" OR "cognitive development" OR "emotional development" OR "psychological development" OR "social development" OR "psychosocial development" OR "identity development" OR "refram*" OR "reappraisal" OR "restor*" OR "reinterpret*" OR "compassio*" OR "empath*" OR "gratitude" OR "grateful" OR "optimis*" OR "satisf*" OR "happiness" OR "peace*" OR "emotional well-being" OR "psychological well-being" OR "mental well-being" OR "social well-being" OR "psychosocial well-being" OR "spiritual well-being" OR "existential well-being" OR "subjective well-being")) ) | 224,300 |
| S9 | S7 OR S8 | 410,555 |
| S10 | ( (MH "Infant+") OR (MH "Child+") OR (MH "Adolescence+") ) AND ( (MH "Self Report+") OR (MH "Self Assessment") OR (MH "Interviews+") OR (MH "Focus Groups") OR (MH "Diaries") OR (MH "Drawing") OR (MH "Autobiographies") OR (MH "Storytelling") OR (MH "Narratives+") OR (MH "Writing") OR (MH "Observational Methods") OR (MH "Action Research") OR (MH "Art Therapy") OR (MH "Play Therapy") OR (MH "Music Therapy") ) | 111,725 |
| S11 | TI ( ("child-led" OR "adolescent-led" OR "youth-led" OR "child-centered" OR "adolescent-centered" OR "youth-centered" OR "child-centred" OR "adolescent-centred" OR "youth-centred" OR "child-focused" OR "adolescent-focused" OR "youth-focused" OR (child* N3 voice*) OR (adolescent* N3 voice*) OR (youth* N3 voice*) OR (child* N3 perspective*) OR (adolescent* N3 perspective*) OR (youth* N3 perspective*)) OR (("newborn*" OR "neonate*" OR "infan*" OR "baby*" OR "babies" OR "toddler*" OR "preschool*" OR "nursery school*" OR "kindergarten*" OR "school-age*" OR "early age*" OR "elementary school*" OR "primary school*" OR "tween*" OR "preteen*" OR "prepuberty" OR "prepubescent*" OR "prepubertal*" OR "child*" OR "middle school*" OR "teen*" OR "puberty" OR "pubescent*" OR "pubertal*" OR "adolescen*" OR "high school*" OR "youth*" OR "pediatric" OR "paediatric") N3 ("self-report*" OR "patient-report*" OR "self-assess*" OR "self-evaluat*" OR "interview*" OR "focus group" OR "diar*" OR "journal*" OR "drawing*" OR "draw-and-tell" OR "draw-and-write" OR "painting*" OR "music*" OR "photo*" OR "storytelling" OR "life stor*" OR "play therapy" OR "expressive writing" OR "observ*" OR "narrative*" OR "participatory" OR "advisory board" OR "autobiography")) ) OR AB ( ("child-led" OR "adolescent-led" OR "youth-led" OR "child-centered" OR "adolescent-centered" OR "youth-centered" OR "child-centred" OR "adolescent-centred" OR "youth-centred" OR "child-focused" OR "adolescent-focused" OR "youth-focused" OR (child* N3 voice*) OR (adolescent* N3 voice*) OR (youth* N3 voice*) OR (child* N3 perspective*) OR (adolescent* N3 perspective*) OR (youth* N3 perspective*)) OR (("newborn*" OR "neonate*" OR "infan*" OR "baby*" OR "babies" OR "toddler*" OR "preschool*" OR "nursery school*" OR "kindergarten*" OR "school-age*" OR "early age*" OR "elementary school*" OR "primary school*" OR "tween*" OR "preteen*" OR "prepuberty" OR "prepubescent*" OR "prepubertal*" OR "child*" OR "middle school*" OR "teen*" OR "puberty" OR "pubescent*" OR "pubertal*" OR "adolescen*" OR "high school*" OR "youth*" OR "pediatric" OR "paediatric") N3 ("self-report*" OR "patient-report*" OR "self-assess*" OR "self-evaluat*" OR "interview*" OR "focus group" OR "diar*" OR "journal*" OR "drawing*" OR "draw-and-tell" OR "draw-and-write" OR "painting*" OR "music*" OR "photo*" OR "storytelling" OR "life stor*" OR "play therapy" OR "expressive writing" OR "observ*" OR "narrative*" OR "participatory" OR "advisory board" OR "autobiography"))) | 46,964 |
| S12 | S10 OR S11 | 145,225 |
| S13 | S3 AND S6 AND S9 AND S12 | 3,333 |

**Table S7**

*Subject Headings for the MEDLINE Database (EBSCOhost)*

| Concept | Subject Headings |
| --- | --- |
| Children and adolescents | (MH "Infant+") OR (MH "Child+") OR (MH "Adolescent") |
| Cancer | (MH "Neoplasms+") OR (MH "Medical Oncology+") OR (MH "Oncology Nursing") OR (MH "Integrative Oncology") OR (MH "Cancer Pain") OR (MH "Cancer Survivors") |
| Meaning-making processes | (MH "Attitude to Health+") OR (MH "Attitude to Death") OR (MH "Religion+") OR (MH "Health Belief Model+") OR (MH "Knowledge") OR (MH "Identity Crisis") OR (MH "Motivation") OR (MH "Sense of Agency") OR (MH "Intention") OR (MH "Goals") OR (MH "Conflict, Psychological+") OR (MH "Aspirations, Psychological") OR (MH "Psychology, Positive") OR (MH "Self Concept+") OR (MH "Life Change Events") OR (MH "Stress, Psychological") OR (MH "Subjective Stress") OR (MH "Adverse Childhood Experiences") OR (MH "Self-Control+") OR (MH "Mental Processes") OR (MH "Cognitive Reflection") OR (MH "Rumination, Cognitive") OR (MH "Metacognition") OR (MH "Comprehension") OR (MH "Cognitive Dissonance") OR (MH "Cognition") OR (MH "Awareness") OR (MH "Perception+") OR (MH "Thinking+") OR (MH "Theory of Mind") OR (MH "Emotions") OR (MH "Psychological Distress") OR (MH "Fear") OR (MH "Hope") OR (MH "Empathy") OR (MH "Optimism") OR (MH "Uncertainty") OR (MH "Coping Skills") OR (MH "Psychological Growth") OR (MH "Posttraumatic Growth, Psychological") OR (MH "Emotional Adjustment+") OR (MH "Adaptation, Psychological") OR (MH "Resilience, Psychological") OR (MH "Child Development") OR (MH "Adolescent Development") OR (MH "Psychological Well-Being") OR (MH "Happiness") |
| Child and adolescent perspective | (MH "Self Report") OR (MH "Interview, Psychological") OR (MH "Interviews as Topic") OR (MH "Focus Groups") OR (MH "Diaries as Topic") OR (MH "Paintings") OR (MH "Photography+") OR (MH "Observation") OR (MH "Narration") OR (MH "Community-Based Participatory Research") OR (MH "Autobiographies as Topic") OR (MH "Art Therapy") OR (MH "Play Therapy") OR (MH "Music Therapy") OR (MH "Writing") |

**Table S8**

*Search Strategy and Results for the MEDLINE Database (EBSCOhost)*

| No. | Query | Search Result |
| --- | --- | --- |
| S1 | (MH "Infant+") OR (MH "Child+") OR (MH "Adolescent") | 4,145,687 |
| S2 | TI ( "newborn*" OR "neonate*" OR "infan*" OR "baby*" OR "babies" OR "toddler*" OR "preschool*" OR "nursery school*" OR "kindergarten*" OR "school-age*" OR "early age*" OR "elementary school*" OR "primary school*" OR "tween*" OR "preteen*" OR "prepuberty" OR "prepubescent*" OR "prepubertal*" OR "child*" OR "middle school*" OR "teen*" OR "puberty" OR "pubescent*" OR "pubertal*" OR "adolescen*" OR "high school*" OR "youth*" OR "pediatric" OR "paediatric" ) OR AB ( "newborn*" OR "neonate*" OR "infan*" OR "baby*" OR "babies" OR "toddler*" OR "preschool*" OR "nursery school*" OR "kindergarten*" OR "school-age*" OR "early age*" OR "elementary school*" OR "primary school*" OR "tween*" OR "preteen*" OR "prepuberty" OR "prepubescent*" OR "prepubertal*" OR "child*" OR "middle school*" OR "teen*" OR "puberty" OR "pubescent*" OR "pubertal*" OR "adolescen*" OR "high school*" OR "youth*" OR "pediatric" OR "paediatric" ) | 2,818,444 |
| S3 | S1 OR S2 | 4,966,617 |
| S4 | (MH "Neoplasms+") OR (MH "Medical Oncology+") OR (MH "Oncology Nursing") OR (MH "Integrative Oncology") OR (MH "Cancer Pain") OR (MH "Cancer Survivors") | 4,098,732 |
| S5 | TI ( "cancer" OR "cancer survivor*" OR "neoplasm*" OR "oncolog*" OR "malignant tumor*" OR "malignant tumour*" OR "leukemia" OR "leukaemia" OR "brain tumor*" OR "brain tumour*"OR "neuroblastoma" OR "wilms tumor*" OR "wilms tumour*" OR "retinoblastoma" OR "yolk sac tumor*" OR "yolk sac tumour*" OR "rhabdomyosarcoma" OR "osteosarcoma" OR "sarcoma*" OR "lymphoma" ) OR AB ( "cancer" OR "cancer survivor*" OR "neoplasm*" OR "oncolog*" OR "malignant tumor*" OR "malignant tumour*" OR "leukemia" OR "leukaemia" OR "brain tumor*" OR "brain tumour*"OR "neuroblastoma" OR "wilms tumor*" OR "wilms tumour*" OR "retinoblastoma" OR "yolk sac tumor*" OR "yolk sac tumour*" OR "rhabdomyosarcoma" OR "osteosarcoma" OR "sarcoma*" OR "lymphoma" ) | 3,166,809 |
| S6 | S4 OR S5 | 4,985,263 |
| S7 | ( (MH "Infant+") OR (MH "Child+") OR (MH "Adolescent") ) AND ( (MH "Attitude to Health+") OR (MH "Attitude to Death") OR (MH "Religion+") OR (MH "Health Belief Model+") OR (MH "Knowledge") OR (MH "Identity Crisis") OR (MH "Motivation") OR (MH "Sense of Agency") OR (MH "Intention") OR (MH "Goals") OR (MH "Conflict, Psychological+") OR (MH "Aspirations, Psychological") OR (MH "Psychology, Positive") OR (MH "Self Concept+") OR (MH "Life Change Events") OR (MH "Stress, Psychological") OR (MH "Subjective Stress") OR (MH "Adverse Childhood Experiences") OR (MH "Self-Control+") OR (MH "Mental Processes") OR (MH "Cognitive Reflection") OR (MH "Rumination, Cognitive") OR (MH "Metacognition") OR (MH "Comprehension") OR (MH "Cognitive Dissonance") OR (MH "Cognition") OR (MH "Awareness") OR (MH "Perception+") OR (MH "Thinking+") OR (MH "Theory of Mind") OR (MH "Emotions") OR (MH "Psychological Distress") OR (MH "Fear") OR (MH "Hope") OR (MH "Empathy") OR (MH "Optimism") OR (MH "Uncertainty") OR (MH "Coping Skills") OR (MH "Psychological Growth") OR (MH "Posttraumatic Growth, Psychological") OR (MH "Emotional Adjustment+") OR (MH "Adaptation, Psychological") OR (MH "Resilience, Psychological") OR (MH "Child Development") OR (MH "Adolescent Development") OR (MH "Psychological Well-Being") OR (MH "Happiness") ) | 500,045 |
| S8 | TI ( ("meaning making" OR "sense making" OR "meaning construction" OR "meaning reconstruction" OR "global meaning" OR "global belief*" OR "situational meaning" OR "positive psycholog*" OR "positive meaning*" OR "positive appraisal*" OR "positive interpretation" OR "positive reappraisal*" OR "positive reinterpretation" OR "appraised meaning" OR "perceived meaning" OR (meaning N3 made) OR "meaning-centered" OR "meaning-centred" OR "meaning-based" OR "meaning-focused" OR "meaning-oriented" OR "meaning-related" OR "meaningful*" OR "meaningless*" OR (meaning N3 violat*) OR "meaning in life" OR (meaning* N3 disease*) OR (meaning* N3 illness*) OR (meaning* N3 cancer*) OR (meaning* N3 survivor*) OR (meaning* N3 patient*) OR (religious N3 meaning*) OR (spiritual N3 meaning*) OR (social N3 meaning*) OR (cultural N3 meaning*) OR (sociocultural N3 meaning*) OR (existential N3 meaning*)) OR (("newborn*" OR "neonate*" OR "infan*" OR "baby*" OR "babies" OR "toddler*" OR "preschool*" OR "nursery school*" OR "kindergarten*" OR "school-age*" OR "early age*" OR "elementary school*" OR "primary school*" OR "tween*" OR "preteen*" OR "prepuberty" OR "prepubescent*" OR "prepubertal*" OR "child*" OR "middle school*" OR "teen*" OR "puberty" OR "pubescent*" OR "pubertal*" OR "adolescen*" OR "high school*" OR "youth*" OR "pediatric" OR "paediatric") N3 ("meaning*" OR "significance" OR "attitude*" OR "belief*" OR "value" OR "worldview" OR "world view" OR "faith*" OR "knowledge" OR "religio*" OR "spiritual*" OR "existen*" OR "identit*" OR "self-concept" OR "fair*" OR "just*" OR "motivat*" OR "goal*" OR "desir*" OR "preference*" OR "sense of meaning*" OR "sense of purpose" OR "sense of self*" OR "sense of agency" OR "sense of connectedness" OR "sense of belonging" OR "lived experience*" OR "stress*" OR "trauma*" OR "advers*" OR "attribut*" OR "appraise*" OR "appraisal*" OR "control*" OR "self-congruence" OR "sense of coherence" OR "self-efficacy" OR "cognitive dissonance" OR "disequilibrium" OR "discrepan*" OR "dissatisf*" OR "distress*" OR "doubt*" OR "fear*" OR "grief" OR "hope*" OR "suffering" OR "uncertain*" OR "anxiety" OR "anxious*" OR "depress*" OR "intrusive thought*" OR "accommodation" OR "assimilation" OR "aware*" OR "concept*" OR "expect*" OR "strateg*" OR "perceive*" OR "perception" OR "reflect*" OR "schema*" OR "coping" OR "understand*" OR "interpret*" OR "comprehen*" OR "emotional* process*" OR "cognitive* process*" OR "adjustment" OR "adaptation" OR "adaptive" OR "acceptance" OR "regulation" OR "resilienc*" OR "post-traumatic growth" OR "cancer-related growth" OR "cognitive development" OR "emotional development" OR "psychological development" OR "social development" OR "psychosocial development" OR "identity development" OR "refram*" OR "reappraisal" OR "restor*" OR "reinterpret*" OR "compassio*" OR "empath*" OR "gratitude" OR "grateful" OR "optimis*" OR "satisf*" OR "happiness" OR "peace*" OR "emotional well-being" OR "psychological well-being" OR "mental well-being" OR "social well-being" OR "psychosocial well-being" OR "spiritual well-being" OR "existential well-being" OR "subjective well-being")) ) OR AB ( ("meaning making" OR "sense making" OR "meaning construction" OR "meaning reconstruction" OR "global meaning" OR "global belief*" OR "situational meaning" OR "positive psycholog*" OR "positive meaning*" OR "positive appraisal*" OR "positive interpretation" OR "positive reappraisal*" OR "positive reinterpretation" OR "appraised meaning" OR "perceived meaning" OR (meaning N3 made) OR "meaning-centered" OR "meaning-centred" OR "meaning-based" OR "meaning-focused" OR "meaning-oriented" OR "meaning-related" OR "meaningful*" OR "meaningless*" OR (meaning N3 violat*) OR "meaning in life" OR (meaning* N3 disease*) OR (meaning* N3 illness*) OR (meaning* N3 cancer*) OR (meaning* N3 survivor*) OR (meaning* N3 patient*) OR (religious N3 meaning*) OR (spiritual N3 meaning*) OR (social N3 meaning*) OR (cultural N3 meaning*) OR (sociocultural N3 meaning*) OR (existential N3 meaning*)) OR (("newborn*" OR "neonate*" OR "infan*" OR "baby*" OR "babies" OR "toddler*" OR "preschool*" OR "nursery school*" OR "kindergarten*" OR "school-age*" OR "early age*" OR "elementary school*" OR "primary school*" OR "tween*" OR "preteen*" OR "prepuberty" OR "prepubescent*" OR "prepubertal*" OR "child*" OR "middle school*" OR "teen*" OR "puberty" OR "pubescent*" OR "pubertal*" OR "adolescen*" OR "high school*" OR "youth*" OR "pediatric" OR "paediatric") N3 ("meaning*" OR "significance" OR "attitude*" OR "belief*" OR "value" OR "worldview" OR "world view" OR "faith*" OR "knowledge" OR "religio*" OR "spiritual*" OR "existen*" OR "identit*" OR "self-concept" OR "fair*" OR "just*" OR "motivat*" OR "goal*" OR "desir*" OR "preference*" OR "sense of meaning*" OR "sense of purpose" OR "sense of self*" OR "sense of agency" OR "sense of connectedness" OR "sense of belonging" OR "lived experience*" OR "stress*" OR "trauma*" OR "advers*" OR "attribut*" OR "appraise*" OR "appraisal*" OR "control*" OR "self-congruence" OR "sense of coherence" OR "self-efficacy" OR "cognitive dissonance" OR "disequilibrium" OR "discrepan*" OR "dissatisf*" OR "distress*" OR "doubt*" OR "fear*" OR "grief" OR "hope*" OR "suffering" OR "uncertain*" OR "anxiety" OR "anxious*" OR "depress*" OR "intrusive thought*" OR "accommodation" OR "assimilation" OR "aware*" OR "concept*" OR "expect*" OR "strateg*" OR "perceive*" OR "perception" OR "reflect*" OR "schema*" OR "coping" OR "understand*" OR "interpret*" OR "comprehen*" OR "emotional* process*" OR "cognitive* process*" OR "adjustment" OR "adaptation" OR "adaptive" OR "acceptance" OR "regulation" OR "resilienc*" OR "post-traumatic growth" OR "cancer-related growth" OR "cognitive development" OR "emotional development" OR "psychological development" OR "social development" OR "psychosocial development" OR "identity development" OR "refram*" OR "reappraisal" OR "restor*" OR "reinterpret*" OR "compassio*" OR "empath*" OR "gratitude" OR "grateful" OR "optimis*" OR "satisf*" OR "happiness" OR "peace*" OR "emotional well-being" OR "psychological well-being" OR "mental well-being" OR "social well-being" OR "psychosocial well-being" OR "spiritual well-being" OR "existential well-being" OR "subjective well-being")) ) | 499,716 |
| S9 | S7 OR S8 | 902,291 |
| S10 | ( (MH "Infant+") OR (MH "Child+") OR (MH "Adolescent") ) AND ( (MH "Self Report") OR (MH "Interview, Psychological") OR (MH "Interviews as Topic") OR (MH "Focus Groups") OR (MH "Diaries as Topic") OR (MH "Paintings") OR (MH "Photography+") OR (MH "Observation") OR (MH "Narration") OR (MH "Community-Based Participatory Research") OR (MH "Autobiographies as Topic") OR (MH "Art Therapy") OR (MH "Play Therapy") OR (MH "Music Therapy") OR (MH "Writing") ) | 161,706 |
| S11 | TI ( ("child-led" OR "adolescent-led" OR "youth-led" OR "child-centered" OR "adolescent-centered" OR "youth-centered" OR "child-centred" OR "adolescent-centred" OR "youth-centred" OR "child-focused" OR "adolescent-focused" OR "youth-focused" OR (child* N3 voice*) OR (adolescent* N3 voice*) OR (youth* N3 voice*) OR (child* N3 perspective*) OR (adolescent* N3 perspective*) OR (youth* N3 perspective*)) OR (("newborn*" OR "neonate*" OR "infan*" OR "baby*" OR "babies" OR "toddler*" OR "preschool*" OR "nursery school*" OR "kindergarten*" OR "school-age*" OR "early age*" OR "elementary school*" OR "primary school*" OR "tween*" OR "preteen*" OR "prepuberty" OR "prepubescent*" OR "prepubertal*" OR "child*" OR "middle school*" OR "teen*" OR "puberty" OR "pubescent*" OR "pubertal*" OR "adolescen*" OR "high school*" OR "youth*" OR "pediatric" OR "paediatric") N3 ("self-report*" OR "patient-report*" OR "self-assess*" OR "self-evaluat*" OR "interview*" OR "focus group" OR "diar*" OR "journal*" OR "drawing*" OR "draw-and-tell" OR "draw-and-write" OR "painting*" OR "music*" OR "photo*" OR "storytelling" OR "life stor*" OR "play therapy" OR "expressive writing" OR "observ*" OR "narrative*" OR "participatory" OR "advisory board" OR "autobiography")) ) OR AB ( ("child-led" OR "adolescent-led" OR "youth-led" OR "child-centered" OR "adolescent-centered" OR "youth-centered" OR "child-centred" OR "adolescent-centred" OR "youth-centred" OR "child-focused" OR "adolescent-focused" OR "youth-focused" OR (child* N3 voice*) OR (adolescent* N3 voice*) OR (youth* N3 voice*) OR (child* N3 perspective*) OR (adolescent* N3 perspective*) OR (youth* N3 perspective*)) OR (("newborn*" OR "neonate*" OR "infan*" OR "baby*" OR "babies" OR "toddler*" OR "preschool*" OR "nursery school*" OR "kindergarten*" OR "school-age*" OR "early age*" OR "elementary school*" OR "primary school*" OR "tween*" OR "preteen*" OR "prepuberty" OR "prepubescent*" OR "prepubertal*" OR "child*" OR "middle school*" OR "teen*" OR "puberty" OR "pubescent*" OR "pubertal*" OR "adolescen*" OR "high school*" OR "youth*" OR "pediatric" OR "paediatric") N3 ("self-report*" OR "patient-report*" OR "self-assess*" OR "self-evaluat*" OR "interview*" OR "focus group" OR "diar*" OR "journal*" OR "drawing*" OR "draw-and-tell" OR "draw-and-write" OR "painting*" OR "music*" OR "photo*" OR "storytelling" OR "life stor*" OR "play therapy" OR "expressive writing" OR "observ*" OR "narrative*" OR "participatory" OR "advisory board" OR "autobiography")) ) | 110,594 |
| S12 | S10 OR S11 | 262,998 |
| S13 | S3 AND S6 AND S9 AND S12 | 3,513 |

**Table S9**

*Search Strategy and Results for the Web of Science Database*

| No. | Query | Search Result |
| --- | --- | --- |
| #1 | (TI=("newborn*" OR "neonate*" OR "infan*" OR "baby*" OR "babies" OR "toddler*" OR "preschool*" OR "nursery school*" OR "kindergarten*" OR "school-age*" OR "early age*" OR "elementary school*" OR "primary school*" OR "tween*" OR "preteen*" OR "prepuberty" OR "prepubescent*" OR "prepubertal*" OR "child*" OR "middle school*" OR "teen*" OR "puberty" OR "pubescent*" OR "pubertal*" OR "adolescen*" OR "high school*" OR "youth*" OR "pediatric" OR "paediatric")) OR AB=("newborn*" OR "neonate*" OR "infan*" OR "baby*" OR "babies" OR "toddler*" OR "preschool*" OR "nursery school*" OR "kindergarten*" OR "school-age*" OR "early age*" OR "elementary school*" OR "primary school*" OR "tween*" OR "preteen*" OR "prepuberty" OR "prepubescent*" OR "prepubertal*" OR "child*" OR "middle school*" OR "teen*" OR "puberty" OR "pubescent*" OR "pubertal*" OR "adolescen*" OR "high school*" OR "youth*" OR "pediatric" OR "paediatric") | 3,742,317 |
| #2 | (TI=("cancer" OR "cancer survivor*" OR "neoplasm*" OR "oncolog*" OR "malignant tumor*" OR "malignant tumour*" OR "leukemia" OR "leukaemia" OR "brain tumor*" OR "brain tumour*"OR "neuroblastoma" OR "wilms tumor*" OR "wilms tumour*" OR "retinoblastoma" OR "yolk sac tumor*" OR "yolk sac tumour*" OR "rhabdomyosarcoma" OR "osteosarcoma" OR "sarcoma*" OR "lymphoma")) OR AB=("cancer" OR "cancer survivor*" OR "neoplasm*" OR "oncolog*" OR "malignant tumor*" OR "malignant tumour*" OR "leukemia" OR "leukaemia" OR "brain tumor*" OR "brain tumour*"OR "neuroblastoma" OR "wilms tumor*" OR "wilms tumour*" OR "retinoblastoma" OR "yolk sac tumor*" OR "yolk sac tumour*" OR "rhabdomyosarcoma" OR "osteosarcoma" OR "sarcoma*" OR "lymphoma") | 3,766,715 |
| #3 | (TI=("meaning making" OR "sense making" OR "meaning construction" OR "meaning reconstruction" OR "global meaning" OR "global belief*" OR "situational meaning" OR "positive psycholog*" OR "positive meaning*" OR "positive appraisal*" OR "positive interpretation" OR "positive reappraisal*" OR "positive reinterpretation" OR "appraised meaning" OR "perceived meaning" OR (meaning NEAR/3 made) OR "meaning-centered" OR "meaning-centred" OR "meaning-based" OR "meaning-focused" OR "meaning-oriented" OR "meaning-related" OR "meaningful*" OR "meaningless*" OR (meaning NEAR/3 violat*) OR "meaning in life" OR (meaning* NEAR/3 disease*) OR (meaning* NEAR/3 illness*) OR (meaning* NEAR/3 cancer*) OR (meaning* NEAR/3 survivor*) OR (meaning* NEAR/3 patient*) OR (religious NEAR/3 meaning*) OR (spiritual NEAR/3 meaning*) OR (social NEAR/3 meaning*) OR (cultural NEAR/3 meaning*) OR (sociocultural NEAR/3 meaning*) OR (existential NEAR/3 meaning*))) OR TI=(("newborn*" OR "neonate*" OR "infan*" OR "baby*" OR "babies" OR "toddler*" OR "preschool*" OR "nursery school*" OR "kindergarten*" OR "school-age*" OR "early age*" OR "elementary school*" OR "primary school*" OR "tween*" OR "preteen*" OR "prepuberty" OR "prepubescent*" OR "prepubertal*" OR "child*" OR "middle school*" OR "teen*" OR "puberty" OR "pubescent*" OR "pubertal*" OR "adolescen*" OR "high school*" OR "youth*" OR "pediatric" OR "paediatric") NEAR/3 ("meaning*" OR "significance" OR "attitude*" OR "belief*" OR "value" OR "worldview" OR "world view" OR "faith*" OR "knowledge" OR "religio*" OR "spiritual*" OR "existen*" OR "identit*" OR "self-concept" OR "fair*" OR "just*" OR "motivat*" OR "goal*" OR "desir*" OR "preference*" OR "sense of meaning*" OR "sense of purpose" OR "sense of self*" OR "sense of agency" OR "sense of connectedness" OR "sense of belonging" OR "lived experience*" OR "stress*" OR "trauma*" OR "advers*" OR "attribut*" OR "appraise*" OR "appraisal*" OR "control*" OR "self-congruence" OR "sense of coherence" OR "self-efficacy" OR "cognitive dissonance" OR "disequilibrium" OR "discrepan*" OR "dissatisf*" OR "distress*" OR "doubt*" OR "fear*" OR "grief" OR "hope*" OR "suffering" OR "uncertain*" OR "anxiety" OR "anxious*" OR "depress*" OR "intrusive thought*" OR "accommodation" OR "assimilation" OR "aware*" OR "concept*" OR "expect*" OR "strateg*" OR "perceive*" OR "perception" OR "reflect*" OR "schema*" OR "coping" OR "understand*" OR "interpret*" OR "comprehen*" OR "emotional* process*" OR "cognitive* process*" OR "adjustment" OR "adaptation" OR "adaptive" OR "acceptance" OR "regulation" OR "resilienc*" OR "post-traumatic growth" OR "cancer-related growth" OR "cognitive development" OR "emotional development" OR "psychological development" OR "social development" OR "psychosocial development" OR "identity development" OR "refram*" OR "reappraisal" OR "restor*" OR "reinterpret*" OR "compassio*" OR "empath*" OR "gratitude" OR "grateful" OR "optimis*" OR "satisf*" OR "happiness" OR "peace*" OR "emotional well-being" OR "psychological well-being" OR "mental well-being" OR "social well-being" OR "psychosocial well-being" OR "spiritual well-being" OR "existential well-being" OR "subjective well-being")) | 212,903 |
| #4 | (AB=("meaning making" OR "sense making" OR "meaning construction" OR "meaning reconstruction" OR "global meaning" OR "global belief*" OR "situational meaning" OR "positive psycholog*" OR "positive meaning*" OR "positive appraisal*" OR "positive interpretation" OR "positive reappraisal*" OR "positive reinterpretation" OR "appraised meaning" OR "perceived meaning" OR (meaning NEAR/3 made) OR "meaning-centered" OR "meaning-centred" OR "meaning-based" OR "meaning-focused" OR "meaning-oriented" OR "meaning-related" OR "meaningful*" OR "meaningless*" OR (meaning NEAR/3 violat*) OR "meaning in life" OR (meaning* NEAR/3 disease*) OR (meaning* NEAR/3 illness*) OR (meaning* NEAR/3 cancer*) OR (meaning* NEAR/3 survivor*) OR (meaning* NEAR/3 patient*) OR (religious NEAR/3 meaning*) OR (spiritual NEAR/3 meaning*) OR (social NEAR/3 meaning*) OR (cultural NEAR/3 meaning*) OR (sociocultural NEAR/3 meaning*) OR (existential NEAR/3 meaning*))) OR AB=(("newborn*" OR "neonate*" OR "infan*" OR "baby*" OR "babies" OR "toddler*" OR "preschool*" OR "nursery school*" OR "kindergarten*" OR "school-age*" OR "early age*" OR "elementary school*" OR "primary school*" OR "tween*" OR "preteen*" OR "prepuberty" OR "prepubescent*" OR "prepubertal*" OR "child*" OR "middle school*" OR "teen*" OR "puberty" OR "pubescent*" OR "pubertal*" OR "adolescen*" OR "high school*" OR "youth*" OR "pediatric" OR "paediatric") NEAR/3 ("meaning*" OR "significance" OR "attitude*" OR "belief*" OR "value" OR "worldview" OR "world view" OR "faith*" OR "knowledge" OR "religio*" OR "spiritual*" OR "existen*" OR "identit*" OR "self-concept" OR "fair*" OR "just*" OR "motivat*" OR "goal*" OR "desir*" OR "preference*" OR "sense of meaning*" OR "sense of purpose" OR "sense of self*" OR "sense of agency" OR "sense of connectedness" OR "sense of belonging" OR "lived experience*" OR "stress*" OR "trauma*" OR "advers*" OR "attribut*" OR "appraise*" OR "appraisal*" OR "control*" OR "self-congruence" OR "sense of coherence" OR "self-efficacy" OR "cognitive dissonance" OR "disequilibrium" OR "discrepan*" OR "dissatisf*" OR "distress*" OR "doubt*" OR "fear*" OR "grief" OR "hope*" OR "suffering" OR "uncertain*" OR "anxiety" OR "anxious*" OR "depress*" OR "intrusive thought*" OR "accommodation" OR "assimilation" OR "aware*" OR "concept*" OR "expect*" OR "strateg*" OR "perceive*" OR "perception" OR "reflect*" OR "schema*" OR "coping" OR "understand*" OR "interpret*" OR "comprehen*" OR "emotional* process*" OR "cognitive* process*" OR "adjustment" OR "adaptation" OR "adaptive" OR "acceptance" OR "regulation" OR "resilienc*" OR "post-traumatic growth" OR "cancer-related growth" OR "cognitive development" OR "emotional development" OR "psychological development" OR "social development" OR "psychosocial development" OR "identity development" OR "refram*" OR "reappraisal" OR "restor*" OR "reinterpret*" OR "compassio*" OR "empath*" OR "gratitude" OR "grateful" OR "optimis*" OR "satisf*" OR "happiness" OR "peace*" OR "emotional well-being" OR "psychological well-being" OR "mental well-being" OR "social well-being" OR "psychosocial well-being" OR "spiritual well-being" OR "existential well-being" OR "subjective well-being")) | 718,442 |
| #5 | #3 OR #4 | 834,320 |
| #6 | (TI=(("child-led" OR "adolescent-led" OR "youth-led" OR "child-centered" OR "adolescent-centered" OR "youth-centered" OR "child-centred" OR "adolescent-centred" OR "youth-centred" OR "child-focused" OR "adolescent-focused" OR "youth-focused" OR (child* NEAR/3 voice*) OR (adolescent* NEAR/3 voice*) OR (youth* NEAR/3 voice*) OR (child* NEAR/3 perspective*) OR (adolescent* NEAR/3 perspective*) OR (youth* NEAR/3 perspective*)) OR (("newborn*" OR "neonate*" OR "infan*" OR "baby*" OR "babies" OR "toddler*" OR "preschool*" OR "nursery school*" OR "kindergarten*" OR "school-age*" OR "early age*" OR "elementary school*" OR "primary school*" OR "tween*" OR "preteen*" OR "prepuberty" OR "prepubescent*" OR "prepubertal*" OR "child*" OR "middle school*" OR "teen*" OR "puberty" OR "pubescent*" OR "pubertal*" OR "adolescen*" OR "high school*" OR "youth*" OR "pediatric" OR "paediatric") NEAR/3 ("self-report*" OR "patient-report*" OR "self-assess*" OR "self-evaluat*" OR "interview*" OR "focus group" OR "diar*" OR "journal*" OR "drawing*" OR "draw-and-tell" OR "draw-and-write" OR "painting*" OR "music*" OR "photo*" OR "storytelling" OR "life stor*" OR "play therapy" OR "expressive writing" OR "observ*" OR "narrative*" OR "participatory" OR "advisory board" OR "autobiography")))) OR AB=(("child-led" OR "adolescent-led" OR "youth-led" OR "child-centered" OR "adolescent-centered" OR "youth-centered" OR "child-centred" OR "adolescent-centred" OR "youth-centred" OR "child-focused" OR "adolescent-focused" OR "youth-focused" OR (child* NEAR/3 voice*) OR (adolescent* NEAR/3 voice*) OR (youth* NEAR/3 voice*) OR (child* NEAR/3 perspective*) OR (adolescent* NEAR/3 perspective*) OR (youth* NEAR/3 perspective*)) OR (("newborn*" OR "neonate*" OR "infan*" OR "baby*" OR "babies" OR "toddler*" OR "preschool*" OR "nursery school*" OR "kindergarten*" OR "school-age*" OR "early age*" OR "elementary school*" OR "primary school*" OR "tween*" OR "preteen*" OR "prepuberty" OR "prepubescent*" OR "prepubertal*" OR "child*" OR "middle school*" OR "teen*" OR "puberty" OR "pubescent*" OR "pubertal*" OR "adolescen*" OR "high school*" OR "youth*" OR "pediatric" OR "paediatric") NEAR/3 ("self-report*" OR "patient-report*" OR "self-assess*" OR "self-evaluat*" OR "interview*" OR "focus group" OR "diar*" OR "journal*" OR "drawing*" OR "draw-and-tell" OR "draw-and-write" OR "painting*" OR "music*" OR "photo*" OR "storytelling" OR "life stor*" OR "play therapy" OR "expressive writing" OR "observ*" OR "narrative*" OR "participatory" OR "advisory board" OR "autobiography"))) | 152,523 |
| #7 | #1 AND #2 AND #5 AND #6 | 1,016 |
